# Supplementary material for: Scaling of sensory information in large neural populations shows signatures of information-limiting correlations
Source: Nat Commun. 2021 Jan 20;12:473. doi: 10.1038/s41467-020-20722-y (PMC7817840; doi:10.1038/s41467-020-20722-y)
Supplement: Supplementary file 1 — Supplementary Information [file 41467_2020_20722_MOESM1_ESM.pdf]

Scaling of sensory information in large neural populations shows signatures of information-limiting correlations Supplementary Information

MohammadMehdi Kafashan, Anna W. Jaffe, Selmaan N. Chettih, Ramon Nogueira, Iñigo Arandia-Romero, Christopher D. Harvey, Rubén Moreno-Bote, and Jan Drugowitsch

Contents

1 Generalized Fisher information **2**
1.1 Definition and properties of linear Fisher information . . . 2
1.2 Generalizing Fisher information beyond fine discrimination . . . 3
1.3 Bias-corrected generalized Fisher information . . . 5
1.4 Variance of bias-corrected generalized Fisher information . . . 6
1.5 Covariance of bias-corrected generalized Fisher information . . . 8

2 Information scaling models **11**
2.1 Linear non-limiting information scaling for large N . . . 11
2.2 Models for $I_{0,N}$ . . . 12
2.3 Impact of measurement noise . . . 13
2.4 Impact of eye movements . . . 13
2.4.1 Impact on population activity moments . . . 14
2.4.2 Impact on information scaling . . . 15

3 Estimating the information scaling moments from neural data **16**
3.1 Generative model and desired moments . . . 16
3.2 Simulating samples from a large, unobserved population . . . 17
3.3 Estimating the mean . . . 17
3.4 Estimating the variance . . . 17
3.5 Estimating the covariance . . . 18

4 Population activity models **20**
4.1 A Gaussian population activity model with limited information . . . 20
4.2 A visual hierarchy population activity model . . . 20

5 Supplementary Tables **22**

6 Supplementary Figures **24**

Supplementary Note 1. Generalized Fisher information

Fisher information quantifies how much neural activity r tells us about a stimulus θ around a particular reference θ_0. As such, it is a measure of fine discrimination performance. Here, we show how *linear* Fisher information relates to Fisher information in general, show how it can be generalized beyond fine discrimination, and describe some properties of this generalization.

1.1 Definition and properties of linear Fisher information

We can derive *linear* Fisher information in two ways [1, 2]. The first is to assume that $p(\mathbf{r}|\theta)$ is a member of the exponential family with linear sufficient statistics. The second is to show that it is the Fisher information that can be extracted with a minimum-variance unbiased linear decoder. We will provide both derivations in turn.

Let us first assume that neural activity r in response to a stimulus θ follows an exponential family distribution with linear sufficient statistics,

$$p(\mathbf{r}|\theta) = g(\theta)\Phi(\mathbf{r})\exp(\mathbf{h}(\theta)^T\mathbf{r}), \tag{1}$$

where

$$g(\theta) = \frac{1}{\int \Phi(\mathbf{r})\exp(\mathbf{h}(\theta)^T\mathbf{r})d\mathbf{r}}, \tag{2}$$

in which $g(\theta)$, $\Phi(\mathbf{r})$, and $\mathbf{h}(\theta)$ are known functions.

The partial derivative with respect to θ of the log-likelihood function, $\frac{\partial}{\partial\theta}\log p(\mathbf{r}|\theta)$, is called the "score" which is given by

$$\frac{\partial}{\partial\theta}\log p(\mathbf{r}|\theta) = \mathbf{h}'^T(\theta)(\mathbf{r}(\theta) - \mathbf{f}(\theta)), \tag{3}$$

where $\mathbf{f}(\theta) = \mathbb{E}\left(\mathbf{r}(\theta)\right)$ is the population activity vector. Note that the first moment of the score function is zero.

The Fisher information can be derived using the variance of the score function [3] which can be written as follows:

$$I(\theta) = \mathbb{E}\left[\left(\frac{\partial}{\partial\theta}\log p(\mathbf{r}|\theta)\right)^2\right] = \mathbf{h}'(\theta)^T\mathbf{\Sigma}(\theta)\mathbf{h}'(\theta), \tag{4}$$

where $\mathbf{\Sigma}(\theta) = \mathbb{E}\left[\left(\mathbf{r}(\theta) - \mathbf{f}(\theta)\right)\left(\mathbf{r}(\theta) - \mathbf{f}(\theta)\right)^T\right]$ is the noise covariance matrix. To express the Fisher information in terms of $\mathbf{f}(\theta)$, we note that

$$\mathbf{f}'(\theta) = \frac{\mathrm{d}}{\mathrm{d}\theta}\int \mathbf{r}p(\mathbf{r}|\theta)d\mathbf{r} = \mathbf{\Sigma}(\theta)\mathbf{h}'(\theta). \tag{5}$$

Thus, we have $\mathbf{h}'(\theta) = \mathbf{\Sigma}^{-1}(\theta)\mathbf{f}'(\theta)$ [4]. Taking this expression to substitute both instances of $\mathbf{h}'(\theta)$ in the Fisher information results in

$$I(\theta) = \mathbf{f}'(\theta)^T\mathbf{\Sigma}^{-1}(\theta)\mathbf{f}'(\theta). \tag{6}$$

To show that linear Fisher information is the information extractable by a minimum-variance unbiased linear decoder, assume that the decoder linearly combines neural activity of neurons with a projection vector w. For fine discrimination task with two close-by stimuli $\theta_1 = \theta_0 - \delta\theta$ and $\theta_2 = \theta_0 + \delta\theta$ with small $\delta\theta$, the unbiased locally linear estimator for $\hat{\theta}$ is given by

$$\hat{\theta} - \theta_0 = \mathbf{w}^T(\mathbf{r} - \mathbf{f}(\theta_0)). \tag{7}$$

The expectation of the right-hand side around θ_0 is $\mathbf{w}^T\left(\langle\mathbf{r}\rangle - \mathbf{f}(\theta_0)\right) = 0$, demonstrating that the estimator is unbiased. Our aim is to find a w that yields a locally unbiased estimate, that is

$$\frac{\mathrm{d}\mathbb{E}_\theta(\hat{\theta})}{\mathrm{d}\theta} = 1, \tag{8}$$

imposing the constraint

$$\mathbf{w}^T\mathbf{f}'(\theta) = 1. \tag{9}$$

To find the minimum variance estimator satisfying this constraint, note that its variance is given by var $\left(\hat{\theta}\right) = \mathbf{w}^T \boldsymbol{\Sigma} \mathbf{w}$, where $\boldsymbol{\Sigma}$ is the noise covariance matrix around θ_0. Therefore, we aim to find

$$\min_{\mathbf{w}} \mathbf{w}^T \boldsymbol{\Sigma} \mathbf{w}, \qquad \text{s.t. } \mathbf{w}^T \mathbf{f}'(\theta) = 1. \tag{10}$$

Using a Lagrange multiplier to solve the constraint optimization for w results in

$$\mathbf{w}^* = \frac{\boldsymbol{\Sigma}^{-1}\mathbf{f}'}{\mathbf{f}'^T \boldsymbol{\Sigma}^{-1}\mathbf{f}'}, \tag{11}$$

with associated estimator variance

$$\text{var}\left(\hat{\theta}\right) = \frac{1}{\mathbf{f}'^T \boldsymbol{\Sigma}^{-1}\mathbf{f}'}. \tag{12}$$

By the Cramér-Rao bound [3], the Fisher information is the inverse of this variance, resulting in

$$I(\theta) = \frac{1}{\text{var}\left(\hat{\theta}\right)} = \mathbf{f}'^T \boldsymbol{\Sigma}^{-1}\mathbf{f}', \tag{13}$$

which matches the previously derived expression for the linear Fisher information. This demonstrates that linear Fisher information can be interpreted in multiple ways: it is either the Fisher information when restricting the distribution of neural activity to the exponential family with linear sufficient statistics (which contains independent-Poisson populations with dense tuning curves, as well as other distributions [4], or the Fisher information that can be extracted with a linear decoder.

1.2 Generalizing Fisher information beyond fine discrimination

Let us generalize the above to coarse discrimination. To do so, assume two classes, C_1 and C_2, which represent a pair of stimulus orientations at θ_1 and θ_2 in the experiment. As before, we will derive generalized Fisher information in two ways. First, we will derive it by making particular distributional assumptions on $p(\mathbf{r}|\theta_1)$ and $p(\mathbf{r}|\theta_2)$. Then, we will derive it from the perspective of optimal linear discrimination.

For the first approach, assume that $p(\mathbf{r}|\theta_j)$ for both $j \in \{1, 2\}$ follows a Gaussian distribution,

$$\begin{aligned} C_1 &: \ p(\mathbf{r}|\theta_1) = \mathcal{N}(\mathbf{r}|\mathbf{f}_1, \boldsymbol{\Sigma}) \\ C_2 &: \ p(\mathbf{r}|\theta_2) = \mathcal{N}(\mathbf{r}|\mathbf{f}_2, \boldsymbol{\Sigma}), \end{aligned} \tag{14}$$

which have different means, but the same covariance matrix. Under the assumption that θ is a random variable (which takes two values, $\theta \in \{\theta_1, \theta_2\}$, in coarse discrimination tasks), it is easy to find a decision rule that minimize the expected Bayes risk [5]. We will denote L_{ij} as the loss of choosing C_j when C_i is correct. Furthermore, we assume a symmetric decision problem with symmetric loss, that is $L_{12} = L_{21}$ and $L_{11} = L_{22}$, a uniform prior $p(C_1) = p(C_2) = 1/2$, and a preference for making correct choices, that is $L_{11} < L_{12}$. In this case, the expected Bayesian risk, $\sum_{i\in\{1,2\}} L_{i\mathcal{D}(\mathbf{r})} p(C_i|\mathbf{r})$, associated with decision rule $\mathcal{D}(\mathbf{r}) \in \{1, 2\}$ is minimized by

$$\mathcal{D}\left(\mathbf{r}\right) = \begin{cases} 2 & \text{if } \Lambda(\mathbf{r}) = \log \frac{p(\mathbf{r}|\theta_2)}{p(\mathbf{r}|\theta_1)} > 0, \\ 1 & \text{otherwise }, \end{cases} \tag{15}$$

where $\Lambda(\mathbf{r})$ is the log-likelihood ratio. For the assumed Gaussian likelihoods, this log-likelihood ratio is given by

$$\Lambda(\mathbf{r}) = (\mathbf{f}_2 - \mathbf{f}_1)^T \boldsymbol{\Sigma}^{-1} (\mathbf{r} - \mathbf{f}_0), \tag{16}$$

where $\mathbf{f}_0 = \frac{1}{2}(\mathbf{f}_1 + \mathbf{f}_2)$, and $\mathbf{f}_j = \mathbb{E}_{\mathbf{r}|\theta_j}(\mathbf{r})$ for $j \in \{1, 2\}$. Letting $\mathbf{w} = \boldsymbol{\Sigma}^{-1}\delta\mathbf{f}$ with $\delta\mathbf{f} = \mathbf{f}_2 - \mathbf{f}_1$, we can rewrite $\Lambda(\mathbf{r})$ as

$$\Lambda(\mathbf{r}) = \mathbf{w}^T (\mathbf{r} - \mathbf{f}_0). \tag{17}$$

In order to identify how likely this decision rule makes the correct choice, observe that $\Lambda(\mathbf{r})$ follows the following distributions under C_1 and C_2,

$$\Lambda(\mathbf{r})|C_1 \sim \mathcal{N}\left(-\frac{1}{2}\mathbf{w}^T\mathbf{\Sigma}\mathbf{w}, \mathbf{w}^T\mathbf{\Sigma}\mathbf{w}\right), \qquad \Lambda(\mathbf{r})|C_2 \sim \mathcal{N}\left(\frac{1}{2}\mathbf{w}^T\mathbf{\Sigma}\mathbf{w}, \mathbf{w}^T\mathbf{\Sigma}\mathbf{w}\right). \tag{18}$$

Therefore, we can find the probability of making a correct choice under $\mathcal{D}(\mathbf{r})$ by

$$p(\text{correct}) = \frac{1}{2}p\left(\Lambda(\mathbf{r}) \leq 0|C_1\right) + \frac{1}{2}p\left(\Lambda(\mathbf{r}) > 0|C_2\right) = \Phi\left(\frac{1}{2}\sqrt{\mathbf{w}^T\mathbf{\Sigma}\mathbf{w}}\right), \tag{19}$$

where $\Phi(\cdot)$ is the cumulative function of the standard normal distribution. After replacing both instances of w by its definition, $\mathbf{w} = \mathbf{\Sigma}^{-1}\delta\mathbf{f}$, $p(\text{correct})$ becomes

$$p(\text{correct}) = \Phi\left(\frac{1}{2}\sqrt{\delta\mathbf{f}^T\mathbf{\Sigma}^{-1}\delta\mathbf{f}}\right). \tag{20}$$

Comparing this expression to Eq. (6) reveals a close similarity which we can utilize to define the generalized linear Fisher information for coarse discrimination tasks by

$$I_g(\theta) = \frac{\delta\mathbf{f}^T\mathbf{\Sigma}^{-1}\delta\mathbf{f}}{\delta\theta^2}, \tag{21}$$

where $\delta\theta = \theta_2 - \theta_1$ is the stimulus difference. It is easy to see that, for small $\delta\theta$, generalized linear Fisher information converges to linear Fisher information,

$$\lim_{\delta\theta\to 0} I_g(\theta) = \lim_{\delta\theta\to 0}\frac{\delta\mathbf{f}^T\mathbf{\Sigma}^{-1}\delta\mathbf{f}}{\delta\theta^2} = \mathbf{f}'^T\mathbf{\Sigma}^{-1}\mathbf{f}' = I(\theta) \tag{22}$$

As the sensitivity index d' [6] in our case is given by $d' = \sqrt{\delta\mathbf{f}^T\mathbf{\Sigma}^{-1}\delta\mathbf{f}}$ [7, 8, 9], the generalized linear Fisher information can be re-expressed in terms of d' by

$$I_g(\theta) = \frac{d'^2}{\delta\theta^2}. \tag{23}$$

This relationship furthermore results in

$$p(\text{correct}) = \Phi\left(\frac{\delta\theta}{2}\sqrt{I_g(\theta)}\right) = \Phi\left(\frac{d'}{2}\right) \tag{24}$$

illustrating the close relationship between $p(\text{correct})$, d', and $I_g(\theta)$.

An alternative derivation for generalized linear Fisher information is through an optimal linear discriminator with less stringent assumptions on the class-conditional distribution. In this second approach, we assume a linear decoder projecting the neural activity to a one-dimensional readout using

$$\hat{\theta} = \mathbf{w}^T\mathbf{r}. \tag{25}$$

To assign an observed neural activity to a class, we just need to place a threshold on the readout $\hat{\theta}$. To do so, we optimize w to maximize the class separation following Fisher's linear discriminant analysis [10], which minimizes the within-class variance while maximizing the between-class variance of r. As before, let $\mathbf{f}_j$ and $\mathbf{\Sigma}_j$ be mean and noise covariance of neural activity in class C_j, but without making any further assumptions about the class-conditional densities $p(\mathbf{r}|C_j)$. We aim to find the w that maximizes the ratio of the between-class variance to the within-class variance, which is formulated as

$$\max_{\mathbf{w}} \frac{\mathbf{w}^T\delta\mathbf{f}\delta\mathbf{f}^T\mathbf{w}}{\mathbf{w}^T\mathbf{\Sigma}\mathbf{w}}, \qquad \text{s.t. } \|\mathbf{w}\|^2 = 1, \tag{26}$$

where $\delta\mathbf{f}\delta\mathbf{f}^T$ is the between-class covariance matrix and $\mathbf{\Sigma}$ is the average within-class covariance matrix given by

$$\mathbf{\Sigma} = \frac{\mathbf{\Sigma}_1 + \mathbf{\Sigma}_2}{2}. \tag{27}$$

Here, we fix $\|\mathrm{w}\|^2 = 1$, as we are interested in the direction of w but not its length. Using a Lagrange multiplier to solve the constraint optimization for w results in

$$\mathbf{w} = \frac{\mathbf{\Sigma}^{-1}\delta\mathbf{f}}{\delta\mathbf{f}^T\mathbf{\Sigma}^{-1}\delta\mathbf{f}}. \tag{28}$$

This yields the direction, w, to best project the neural activity into one dimension.

To find the associated $p(\text{correct})$, note that $\hat{\theta}$ is the sum of a (potentially large) set of random variables. These random variables are correlated, such that the central limit theorem does not directly apply. Nonetheless, we assume this sum to be approximately Gaussian for both $\hat{\theta}|C_1$ and $\hat{\theta}|C_2$, and given by

$$\hat{\theta}|C_1 \sim \mathcal{N}\left(\mathbf{w}^T\mathbf{f_1}, \mathbf{w}^T\mathbf{\Sigma}_1\mathbf{w}\right), \qquad \hat{\theta}|C_2 \sim \mathcal{N}\left(\mathbf{w}^T\mathbf{f_2}, \mathbf{w}^T\mathbf{\Sigma}_2\mathbf{w}\right). \tag{29}$$

This results in the sensitivity index, d', to be given by

$$d' = \frac{\mathbf{w}^T\mathbf{f_2} - \mathbf{w}^T\mathbf{f_1}}{\sqrt{\frac{1}{2}(\mathbf{w}^T\mathbf{\Sigma}_1\mathbf{w} + \mathbf{w}^T\mathbf{\Sigma}_2\mathbf{w})}} = \frac{\mathbf{w}^T\delta\mathbf{f}}{\sqrt{\mathbf{w}^T\mathbf{\Sigma}\mathbf{w}}} = \sqrt{\delta\mathbf{f}^T\mathbf{\Sigma}^{-1}\delta\mathbf{f}}, \tag{30}$$

yielding the same expression as before. This makes it straightforward to derive the generalized Fisher information as before.

1.3 Bias-corrected generalized Fisher information

Evaluating the generalized Fisher information, Eq. (21), by replacing $\delta\mathbf{f}$ and $\mathbf{\Sigma}$ by its empirical moments estimated from neural data with a limited number of trials leads to biased estimates [11]. In [11], they provide a bias correction for standard Fisher information, but it is unclear if this bias correction also applies to our generalization of Fisher information. In this section, we will derive such a bias correction for our generalization. This correction turns out to be the same as that provided by [11]. This is unsurprising in hindsight, as [11] do not restrict the size of $\delta\theta$ in their derivation, such that it applies to both fine and coarse discrimination.

We assume neural activity $\mathbf{r}_j^t,\ j = 1, 2$ in response to stimulus θ_j in trials $t = 1, ..., T$ to follow a multivariate Gaussian distribution given by

$$\boldsymbol{r}_j^t \sim \mathcal{N}\left(\mathbf{f}_j, \mathbf{\Sigma}\right), \qquad j = 1, 2, \tag{31}$$

where we assume the same covariance matrix for neural activity in response to θ_1 and θ_2. This is not a restriction, as our above derivation from the perspective of a linear discriminator has shown that, if these covariances differ, we can replace them by their average (which is what we do in practice, see below). Under this assumption, the empirical mean and covariance over T trials for each stimulus is distributed as [12]

$$\mu_j = \frac{1}{T}\sum_{t=1}^{T}\boldsymbol{r}_j^t \sim \mathcal{N}\left(\mathbf{f}_j, \frac{\mathbf{\Sigma}}{T}\right), \qquad \mathbf{S}_j = \frac{1}{T-1}\sum_{t=1}^{T}(\boldsymbol{r}_j^t - \mu_j)(\boldsymbol{r}_j^t - \mu_j)^T \sim \mathcal{W}\left(\frac{\mathbf{\Sigma}}{T-1}, T-1\right), \tag{32}$$

where $\mathcal{W}(V_{p\times p}, n)$ is the p-dimensional Wishart distribution with n degrees of freedom.

The naïve estimation of generalized Fisher information, Eq. (21), is obtained by replacing $\delta\mathbf{f}$ and $\mathbf{\Sigma}$ with their unbiased estimates, $\delta\mu$ and $\mathbf{S}$, given by

$$\delta\mu = \mu_1 - \mu_2 \sim \mathcal{N}\left(\delta\mathbf{f}, \frac{2\mathbf{\Sigma}}{T}\right), \qquad \mathbf{S} = \frac{1}{2}(\mathbf{S}_1 + \mathbf{S}_2) \sim \mathcal{W}\left(\frac{\mathbf{\Sigma}}{2(T-1)}, 2(T-1)\right), \tag{33}$$

where $\mathbb{E}(\delta\mu) = \delta\mathbf{f}$ and $\mathbb{E}(\mathbf{S}) = \mathbf{\Sigma}$. Furthermore, the inverse of sample covariance, $\mathbf{S}^{-1}$, follows an inverse Wishart distribution [12] given by

$$\mathbf{S}^{-1} \sim \mathcal{W}^{-1}\left(2(T-1)\mathbf{\Sigma}^{-1}, 2(T-1)\right), \tag{34}$$

which has mean

$$\mathbb{E}(\mathbf{S}^{-1}) = \frac{2(T-1)}{2T-N-3}\mathbf{\Sigma}^{-1} \tag{35}$$

Replacing δf and $\mathbf{\Sigma}$ with $\delta\mu$ and S in Eq. (21) results in the following naive estimator of the generalized Fisher information to be given by

$$\hat{I}_{g,nv}(\theta) = \frac{\delta\mu^T\mathbf{S}^{-1}\delta\mu}{\delta\theta^2}. \tag{36}$$

To evaluate the bias of $I_{g,nv}$, we utilize the fact that the sample mean and sample covariance of Gaussian distributions are independent [12], such that we can express the first moment of $I_{g,nv}$ by

$$\mathbb{E}\left(\hat{I}_{g,nv}\right) = \frac{\mathbb{E}_{\delta\mu,\mathbf{S}}\left(\delta\mu^T\mathbf{S}^{-1}\delta\mu\right)}{\delta\theta^2}, \tag{37}$$

where

$$\begin{aligned}
\mathbb{E}_{\delta\mu,\mathbf{S}}\left(\delta\mu^T\mathbf{S}^{-1}\delta\mu\right) &= \mathbb{E}_{\delta\mu,\mathbf{S}}\left(\mathrm{Tr}\left(\delta\mu\delta\mu^T\mathbf{S}^{-1}\right)\right)\\
&= \mathrm{Tr}\left(\mathbb{E}_{\delta\mu,\mathbf{S}}\left(\delta\mu\delta\mu^T\mathbf{S}^{-1}\right)\right)\\
&= \mathrm{Tr}\left(\mathbb{E}_{\delta\mu}\left(\delta\mu\delta\mu^T\right)\mathbb{E}_{\mathbf{S}}\left(\mathbf{S}^{-1}\right)\right)\\
&= \mathrm{Tr}\left(\left(\delta\mathbf{f}\delta\mathbf{f}^T + \frac{2\mathbf{\Sigma}}{T}\right)\left(\frac{2(T-1)}{2T-N-3}\mathbf{\Sigma}^{-1}\right)\right)\\
&= \frac{2(T-1)}{2T-N-3}\left(\mathrm{Tr}\left(\delta\mathbf{f}\delta\mathbf{f}^T\mathbf{\Sigma}^{-1}\right) + \frac{2N}{T}\right)\\
&= \frac{2(T-1)}{2T-N-3}\left(\delta\mathbf{f}^T\mathbf{\Sigma}^{-1}\delta\mathbf{f} + \frac{2N}{T}\right)\\
&= \frac{2(T-1)}{2T-N-3}\left(I_g\delta\theta^2 + \frac{2N}{T}\right).
\end{aligned} \tag{38}$$

Having the first moment of $\hat{I}_{g,nv}$, we can obtain the expression for the bias-corrected generalized Fisher information, $\hat{I}_{g,bc}$, given by

$$\hat{I}_{g,bc} = \frac{2T-N-3}{2(T-1)}\frac{\delta\mu^T\mathbf{S}^{-1}\delta\mu}{\delta\theta^2} - \frac{2N}{T\delta\theta^2}. \tag{39}$$

This estimate is the same as provided by [11], and will, in expectation, equal the true Fisher information, that is, $\mathbb{E}\left(\hat{I}_{g,bc}\right) = I_g$.

1.4 Variance of bias-corrected generalized Fisher information

Let us now consider the variance of the bias-corrected generalized Fisher information across different draws of T trial/samples from the same neural population. This variance has already been computed by [11], but only as a function of the true information, I_g, which is an unknown quantity. Here, we re-derive this expression for completeness, and additionally derive an unbiased estimated thereof as a function of $\hat{I}_{g,bc}$, which can be computed from experimental data.

The variance of $\hat{I}_{g,bc}$ is given by

$$\mathrm{var}\left(\hat{I}_{g,bc}\right) = \frac{(2T-N-3)^2}{4(T-1)^2\delta\theta^4}\mathrm{var}(\delta\mu^T\mathbf{S}^{-1}\delta\mu), \tag{40}$$

where $\mathrm{var}\left(\delta\mu^T\mathbf{S}^{-1}\delta\mu\right)$ can be decomposed into

$$\mathrm{var}(\delta\mu^T\mathbf{S}^{-1}\delta\mu) = \mathbb{E}\left((\delta\mu^T\mathbf{S}^{-1}\delta\mu)^2\right) - \mathbb{E}\left(\delta\mu^T\mathbf{S}^{-1}\delta\mu\right)^2. \tag{41}$$

The first term in Eq. (41) can be expressed as

$$\begin{aligned}\mathbb{E}\left((\delta\mu^T\mathbf{S}^{-1}\delta\mu)^2\right) &= \mathbb{E}\left(\delta\mu^T\mathbf{S}^{-1}\delta\mu\delta\mu^T\mathbf{S}^{-1}\delta\mu\right)\\ &= \mathbb{E}_{\delta\mu}\left(\delta\mu^T\mathbb{E}_{\mathbf{S}}\left(\mathbf{S}^{-1}\delta\mu\delta\mu^T\mathbf{S}^{-1}\right)\delta\mu\right)\\ &= \frac{4(T-1)^2}{(2T-N-3)(2T-N-5)}\mathbb{E}_{\delta\mu}\left(\delta\mu^T\mathbf{\Sigma}^{-1}\delta\mu\delta\mu^T\mathbf{\Sigma}^{-1}\delta\mu\right)\\ &= \frac{4(T-1)^2}{(2T-N-3)(2T-N-5)}\mathbb{E}_{\delta\mu}\left((\delta\mu^T\mathbf{\Sigma}^{-1}\delta\mu)^2\right)\\ &= \frac{4(T-1)^2}{(2T-N-3)(2T-N-5)}\left(\mathrm{var}(\delta\mu^T\mathbf{\Sigma}^{-1}\delta\mu)+\mathbb{E}_{\delta\mu}\left(\delta\mu^T\mathbf{\Sigma}^{-1}\delta\mu\right)^2\right).\end{aligned} \tag{42}$$

The second term in Eq. (41) can be expressed as

$$\mathbb{E}\left(\delta\mu^T\mathbf{S}^{-1}\delta\mu\right)^2 = \frac{4(T-1)^2}{(2T-N-3)^2}\mathbb{E}_{\delta\mu}\left(\delta\mu^T\mathbf{\Sigma}^{-1}\delta\mu\right)^2. \tag{43}$$

Together, this results in Eq. (41) to be given by

$$\mathrm{var}(\delta\mu^T\mathbf{S}^{-1}\delta\mu) = \frac{4(T-1)^2}{(2T-N-3)(2T-N-5)}\left(\mathrm{var}(\delta\mu^T\mathbf{\Sigma}^{-1}\delta\mu)+\frac{2}{2T-N-3}\mathbb{E}_{\delta\mu}\left(\delta\mu^T\mathbf{\Sigma}^{-1}\delta\mu\right)^2\right). \tag{44}$$

Therefore, $\mathrm{var}(I_{g,bc})$ can be simplified to

$$\mathrm{var}\left(\hat{I}_{g,bc}\right) = \frac{2}{2T-N-5}\left(\frac{2T-N-3}{2\delta\theta^4}\mathrm{var}(\delta\mu^T\mathbf{\Sigma}^{-1}\delta\mu)+\frac{1}{\delta\theta^4}\mathbb{E}_{\delta\mu}\left(\delta\mu^T\mathbf{\Sigma}^{-1}\delta\mu\right)^2\right). \tag{45}$$

To simplify this expression, note that if $\epsilon \sim \mathcal{N}(\mu,\mathbf{\Sigma})$, then, for a constant matrix $\mathbf{\Lambda}$, we have

$$\mathbb{E}(\epsilon^T\mathbf{\Lambda}\epsilon) = \mathrm{Tr}(\mathbf{\Lambda\Sigma}) + \mu^T\mathbf{\Lambda}\mu. \tag{46}$$

Additionally, for a symmetric matrix $\mathbf{\Lambda}$, the variance of the quadratic form is expressed as

$$\mathrm{var}(\epsilon^T\mathbf{\Lambda}\epsilon) = 2\,\mathrm{Tr}\left(\mathbf{\Lambda\Sigma\Lambda\Sigma}\right) + 4\mu^T\mathbf{\Lambda\Sigma\Lambda}\mu. \tag{47}$$

Applying Eqs. (46) and (47) yields

$$\mathrm{var}(\delta\mu^T\mathbf{\Sigma}^{-1}\delta\mu) = \frac{8N}{T^2}+\frac{8}{T}\delta\theta^2 I_g, \qquad \mathbb{E}_{\delta\mu}\left(\delta\mu^T\mathbf{\Sigma}^{-1}\delta\mu\right)^2 = \frac{4N^2}{T^2}+\frac{4N}{T}\delta\theta^2 I_g+\delta\theta^4 I_g^2. \tag{48}$$

Using these expressions results in the final variance

$$\mathrm{var}\left(\hat{I}_{g,bc}\right) = \frac{2}{2T-N-5}\left(I_g^2+\frac{4(2T-3)}{T\delta\theta^2}I_g+\frac{4N(2T-3)}{T^2\delta\theta^4}\right) \tag{49}$$

This is the expression provided by [11]. Unfortunately, it is a function of the true information I_g, which is unknown, such that the variance cannot be evaluated from data.

To find an unbiased estimate of this variance, note that the true information, I_g, shows up as I_g and I_g^2. We already have an unbiased estimate of I_g, and will now derive such an unbiased estimate for I_g^2. Let us denote this estimate by $\left(\hat{I_g^2}\right)_{bc}$ (in contrast to the squared $\hat{I}_{g,bc}$, which is $\hat{I}_{g,bc}^2$). We find it by

$$\begin{aligned}\mathbb{E}\left((\hat{I}_{g,bc})^2\right) &= \mathrm{var}\left(\hat{I}_{g,bc}\right)+\mathbb{E}\left(\hat{I}_{g,bc}\right)^2\\ &= \frac{2}{2T-N-5}\left(I_g^2+\frac{4(2T-3)}{T\delta\theta^2}I_g+\frac{4N(2T-3)}{T^2\delta\theta^4}\right)+I_g^2\\ &= \frac{1}{2T-N-5}\left((2T-N-3)I_g^2+\frac{8(2T-3)}{T\delta\theta^2}I_g+\frac{8N(2T-3)}{T^2\delta\theta^4}\right).\end{aligned} \tag{50}$$

Solving for I_g^2 and substituting I_g by its bias-corrected estimate $\hat{I}_{g,bc}$ reveals the bias-corrected estimate

$$\left(\hat{I_g^2}\right)_{bc} = \frac{2T-N-5}{2T-N-3}\hat{I}_{g,bc}^2 - \frac{1}{2T-N-3}\frac{8(2T-3)}{T\delta\theta^2}\hat{I}_{g,bc} - \frac{1}{2T-N-3}\frac{8N(2T-3)}{T^2\delta\theta^4}, \tag{51}$$

which satisfies $\mathbb{E}\left(\left(\hat{I_g^2}\right)_{bc}\right) = I_g^2$. Substituting the bias corrected estimates of I_g and I_g^2 into Eq. (49) results after some algebra in the unbiased variance estimate

$$\text{var}\left(\hat{I}_{g,bc}\right) = \frac{2}{2T-N-3}\left(\hat{I}_{g,bc}^2 + \frac{4(2T-3)}{T\delta\theta^2}\hat{I}_{g,bc} + \frac{4N(2T-3)}{T^2\delta\theta^4}\right), \tag{52}$$

which can be computed from data.

1.5 Covariance of bias-corrected generalized Fisher information

As we are interested in how information scales with population size, we also need to know how information estimates for different subpopulations relate to each other. Knowing this relationship is essential to our model fits, as fitting the information scaling models to information estimates that are correlated across different population sizes could results in significant mis-estimates if these correlations are ignored. In fact, we will use the results from this section to show in Sec. 3.5 that the increase in information due to adding one more neuron to a population is uncorrelated across different subpopulations. Based on this insight, we thus fitted these information increases rather than absolute informations, as illustrated in Supplementary Figure 4 in the main text.

To identify the relation between the information estimates for different subpopulations, we will focus on two subpopulations with N_x and N_y neurons ($N_y \leq N_x$) where the latter consists of a subset of neurons of the former. That is, the subpopulation with N_x neurons contains all of the N_y neurons in the (possibly) smaller subpopulation. We are interested in how their information estimates co-vary if we estimate both information measures from the same set of T trials.

To find this covariance, let us decompose the true (i.e., non-empirical) moments of the larger subpopulation into

$$\delta\mathbf{f}_x = \begin{pmatrix}\delta\mathbf{f}_y \\ \delta\mathbf{f}_z\end{pmatrix}, \qquad \mathbf{\Sigma}_x = \begin{pmatrix}\mathbf{\Sigma}_y & \mathbf{\Sigma}_u \\ \mathbf{\Sigma}_u^T & \mathbf{\Sigma}_z\end{pmatrix}. \tag{53}$$

Here, $\delta\mathbf{f}_x$ and $\delta\mathbf{f}_y$ are the population tuning differences of the larger and smaller subpopulation, respectively, and we have ordered the neurons in the larger subpopulation such that it contains all shared neurons first, followed by all non-shared neurons. This re-ordering is possible, as the information estimates are independent or how neurons are ordered within a population. Furthermore, $\mathbf{\Sigma}_x$ and $\mathbf{\Sigma}_y$ are the noise covariance matrices of the larger and smaller subpopulation, and $\mathbf{\Sigma}_u$ is the the covariance of shared with non-shared neurons.

Experimentally, we cannot directly observe these moments, but instead estimate them through the empirical moments,

$$\delta\mu_x = \begin{pmatrix}\delta\mu_y \\ \delta\mu_z.\end{pmatrix}, \qquad \mathbf{S}_x = \begin{pmatrix}\mathbf{S}_y & \mathbf{S}_u \\ \mathbf{S}_u^T & \mathbf{S}_z.\end{pmatrix} \tag{54}$$

Using the same properties as in the previous section, these empirical moments relate to the true moments by

$$\delta\mu_x \sim \mathcal{N}\left(\delta\mathbf{f}_x, \frac{2}{T}\mathbf{\Sigma}_x\right), \qquad \mathbf{S}_x^{-1} \sim \mathcal{W}^{-1}\left(2(T-1)\mathbf{\Sigma}_x^{-1}, 2(T-1)\right), \tag{55}$$

$$\delta\mu_y \sim \mathcal{N}\left(\delta\mathbf{f}_y, \frac{2}{T}\mathbf{\Sigma}_y\right), \qquad \mathbf{S}_y^{-1} \sim \mathcal{W}^{-1}\left(2(T-1)\mathbf{\Sigma}_y^{-1}, 2(T-1)\right), \tag{56}$$

The empirical covariances additionally have the properties [13]

$$(\mathbf{S}_z - \mathbf{S}_u\mathbf{S}_y^{-1}\mathbf{S}_u^T)^{-1} \sim \mathcal{W}^{-1}\left(2(T-1)(\mathbf{\Sigma}_z - \mathbf{\Sigma}_u\mathbf{\Sigma}_y^{-1}\mathbf{\Sigma}_u^T)^{-1}, 2(T-1)\right), \tag{57}$$

$$\mathbf{S}_u\mathbf{S}_y^{-1}|\mathbf{S}_y^{-1} \sim \mathcal{MN}_{N_y\times(N_x-N_y)}\left(\mathbf{\Sigma}_u\mathbf{\Sigma}_y^{-1}, \frac{1}{2(T-1)}(\mathbf{\Sigma}_x - \mathbf{\Sigma}_u\mathbf{\Sigma}_y^{-1}\mathbf{\Sigma}_u^T), \mathbf{S}_y^{-1}\right), \tag{58}$$

where $\mathcal{MN}$ is the matrix-normal distribution.

From Eq. (39), the bias-corrected generalized information for two subpopulations denoted as $I^x_{g,bc}$ and $I^y_{g,bc}$ can be written as

$$\hat{I}^x_{g,bc} = \frac{2T - N_x - 3}{2T - 2}\frac{\delta\mu_x^T \mathbf{S}_x^{-1}\delta\mu_x}{\delta\theta^2} - \frac{2N_x}{T\delta\theta^2}, \tag{59}$$

$$\hat{I}^y_{g,bc} = \frac{2T - N_y - 3}{2T - 2}\frac{\delta\mu_y^T \mathbf{S}_y^{-1}\delta\mu_y}{\delta\theta^2} - \frac{2N_y}{T\delta\theta^2}. \tag{60}$$

We can decompose $\hat{I}^x_{g,bc}$ into two terms. The first term is the shared information which is common between subpopulations x and y as both of them contains all of neurons in subpopulation y. The second term is the information gain that is gained by adding the non-shared neurons. This decomposition can be expressed as

$$\hat{I}^x_{g,bc} = \hat{I}^y_{g,bc} + \delta\hat{I}^{x-y}_{g,bc}, \tag{61}$$

where $\delta\hat{I}^{x-y}_{g,bc}$ is the information gain due to the non-shared components between subpopulations x and y. The covariance of $\hat{I}^x_{g,bc}$ and $\hat{I}^y_{g,bc}$ is given by

$$\text{cov}\left(\hat{I}^x_{g,bc}, \hat{I}^y_{g,bc}\right) = \text{var}\left(\hat{I}^y_{g,bc}\right) + \text{cov}\left(\hat{I}^y_{g,bc}, \delta\hat{I}^{x-y}_{g,bc}\right), \tag{62}$$

where we already have expression for the variance on the right-hand side (i.e., Eq. (52)), and only need to find an expression for the covariance.

To calculate $\text{cov}\left(\hat{I}^y_{g,bc}, \delta\hat{I}^{x-y}_{g,bc}\right)$, let us first find an expression for $\delta\hat{I}^{x-y}_{g,bc}$. To find this expression, note that, by the decomposition of $\delta\mu_x$ and $\mathbf{S}_x$, and using block matrix inversion,

$$\delta\mu_x^T \mathbf{S}_x^{-1}\delta\mu_x = \delta\mu_y^T \mathbf{S}_y^{-1}\delta\mu_y + \left(\delta\mu_z - \mathbf{S}_u\mathbf{S}_y^{-1}\delta\mu_y\right)^T \left(\mathbf{S}_z - \mathbf{S}_u\mathbf{S}_y^{-1}\mathbf{S}_u^T\right)^{-1}\left(\delta\mu_z - \mathbf{S}_u\mathbf{S}_y^{-1}\delta\mu_y\right), \tag{63}$$

Substituting this relationship into Eqs. (59) and (60) results in the bias-corrected information gain

$$\begin{aligned}\delta\hat{I}^{x-y}_{g,bc} &= \hat{I}^x_{g,bc} - \hat{I}^y_{g,bc} \\ &= \frac{N_y - N_x}{2T - N_y - 3}\hat{I}^y_{g,bc} + \frac{2T - N_x - 3}{2T - 2}\frac{\left(\delta\mu_z - \mathbf{S}_u\mathbf{S}_y^{-1}\delta\mu_y\right)^T \left(\mathbf{S}_z - \mathbf{S}_u\mathbf{S}_y^{-1}\mathbf{S}_u^T\right)^{-1}\left(\delta\mu_z - \mathbf{S}_u\mathbf{S}_y^{-1}\delta\mu_y\right)}{\delta\theta^2} + \text{const},\end{aligned} \tag{64}$$

where "const" captures all non-stochastic terms that do not contribute to the covariance. Overall, this results in

$$\begin{aligned}\text{cov}\left(\hat{I}^y_{g,bc}, \delta\hat{I}^{x-y}_{g,bc}\right) = &\frac{N_y - N_x}{2T - N_y - 3}\text{var}\left(\hat{I}^y_{g,bc}\right) + \frac{(2T - N_x - 3)(2T - N_y - 3)}{(2T-2)^2\delta\theta^4} \\ &\times \text{cov}\left(\delta\mu_y^T \mathbf{S}_y^{-1}\delta\mu_y, \left(\delta\mu_z - \mathbf{S}_u\mathbf{S}_y^{-1}\delta\mu_y\right)^T \left(\mathbf{S}_z - \mathbf{S}_u\mathbf{S}_y^{-1}\mathbf{S}_u^T\right)^{-1}\left(\delta\mu_z - \mathbf{S}_u\mathbf{S}_y^{-1}\delta\mu_y\right)\right),\end{aligned} \tag{65}$$

where we have substituted Eq. (60) for $I^y_{g,bc}$ to find the second term on the right-hand side. The first term of Eq. (65) is known from Eq. (52). The covariance expression in the second term can be expressed as

$$\begin{aligned}&\text{cov}\left(\delta\mu_y^T \mathbf{S}_y^{-1}\delta\mu_y, \left(\delta\mu_z - \mathbf{S}_u\mathbf{S}_y^{-1}\delta\mu_y\right)^T \left(\mathbf{S}_z - \mathbf{S}_u\mathbf{S}_y^{-1}\mathbf{S}_u^T\right)^{-1}\left(\delta\mu_z - \mathbf{S}_u\mathbf{S}_y^{-1}\delta\mu_y\right)\right) \\ &\quad = \mathbb{E}\left(\delta\mu_y^T \mathbf{S}_y^{-1}\delta\mu_y \left(\delta\mu_z - \mathbf{S}_u\mathbf{S}_y^{-1}\delta\mu_y\right)^T \left(\mathbf{S}_z - \mathbf{S}_u\mathbf{S}_y^{-1}\mathbf{S}_u^T\right)^{-1}\left(\delta\mu_z - \mathbf{S}_u\mathbf{S}_y^{-1}\delta\mu_y\right)\right) \\ &\quad\quad - \mathbb{E}\left(\delta\mu_y^T \mathbf{S}_y^{-1}\delta\mu_y\right)\mathbb{E}\left(\left(\delta\mu_z - \mathbf{S}_u\mathbf{S}_y^{-1}\delta\mu_y\right)^T \left(\mathbf{S}_z - \mathbf{S}_u\mathbf{S}_y^{-1}\mathbf{S}_u^T\right)^{-1}\left(\delta\mu_z - \mathbf{S}_u\mathbf{S}_y^{-1}\delta\mu_y\right)\right)\end{aligned} \tag{66}$$

First we evaluate the last expectation in Eq. (66) which is

$$\mathbb{E}\left(\left(\delta\mu_z - \mathbf{S}_u\mathbf{S}_y^{-1}\delta\mu_y\right)^T \left(\mathbf{S}_z - \mathbf{S}_u\mathbf{S}_y^{-1}\mathbf{S}_u^T\right)^{-1}\left(\delta\mu_z - \mathbf{S}_u\mathbf{S}_y^{-1}\delta\mu_y\right)\right) \tag{67}$$

Conditioned on $\mathbf{S}_y^{-1}$, we observe that $(\mathbf{S}_z - \mathbf{S}_u\mathbf{S}_y^{-1}\mathbf{S}_u^T)^{-1}$ is independent of $\mathbf{S}_u\mathbf{S}_y^{-1}$ [13]. Thus we can first take the expectation of $(\mathbf{S}_z - \mathbf{S}_u\mathbf{S}_y^{-1}\mathbf{S}_u^T)^{-1}$ to get

$$\frac{2T-2}{2T-N_x-3}\left(\delta\mu_z - \mathbf{S}_u\mathbf{S}_y^{-1}\delta\mu_y\right)^T\left(\boldsymbol{\Sigma}_z - \boldsymbol{\Sigma}_u\boldsymbol{\Sigma}_y^{-1}\boldsymbol{\Sigma}_u^T\right)^{-1}\left(\delta\mu_z - \mathbf{S}_u\mathbf{S}_y^{-1}\delta\mu_y\right). \tag{68}$$

Next, we observe that $\mathbf{S}_u\mathbf{S}_y^{-1}|\mathbf{S}_y^{-1}$ is matrix normal, which has a simple expression for the expectation of its quadratic form. Using this expression yields

$$\frac{2T-2}{2T-N_x-3}\left(\delta\mu_z - \boldsymbol{\Sigma}_u\boldsymbol{\Sigma}_y^{-1}\delta\mu_y\right)^T\left(\boldsymbol{\Sigma}_z - \boldsymbol{\Sigma}_u\boldsymbol{\Sigma}_y^{-1}\boldsymbol{\Sigma}_u^T\right)^{-1}\left(\delta\mu_z - \boldsymbol{\Sigma}_u\boldsymbol{\Sigma}_y^{-1}\delta\mu_y\right) + \frac{N_x-N_y}{2T-N_x-3}\delta\mu_y^T\mathbf{S}_y^{-1}\delta\mu_y \tag{69}$$

We do not need to complete the expectation over the remaining random variables because most involved terms cancel out each other later on.

Utilizing the same strategy we evaluate the expectation of the first term in Eq. (66) which is given by

$$\mathbb{E}\left(\delta\mu_y^T\mathbf{S}_y^{-1}\delta\mu_y\left(\delta\mu_z - \mathbf{S}_u\mathbf{S}_y^{-1}\delta\mu_y\right)^T\left(\mathbf{S}_z - \mathbf{S}_u\mathbf{S}_y^{-1}\mathbf{S}_u^T\right)^{-1}\left(\delta\mu_z - \mathbf{S}_u\mathbf{S}_y^{-1}\delta\mu_y\right)\right). \tag{70}$$

Its expectation with respect to $\left(\mathbf{S}_z - \mathbf{S}_u\mathbf{S}_y^{-1}\mathbf{S}_u^T\right)^{-1}$ is

$$\frac{2T-2}{2T-N-3}\delta\mu_y^T\mathbf{S}_y^{-1}\delta\mu_y\left(\delta\mu_z - \mathbf{S}_u\mathbf{S}_y^{-1}\delta\mu_y\right)^T\left(\boldsymbol{\Sigma}_z - \boldsymbol{\Sigma}_u\boldsymbol{\Sigma}_y^{-1}\boldsymbol{\Sigma}_u^T\right)^{-1}\left(\delta\mu_z - \mathbf{S}_u\mathbf{S}_y^{-1}\delta\mu_y\right). \tag{71}$$

The expectation with respect to $\mathbf{S}_u\mathbf{S}_y^{-1}|\mathbf{S}_y^{-1}$ is given by

$$\frac{2T-2}{2T-N_x-3}\delta\mu_y^T\mathbf{S}_y^{-1}\delta\mu_y\left(\delta\mu_z - \boldsymbol{\Sigma}_u\boldsymbol{\Sigma}_y^{-1}\delta\mu_y\right)^T\left(\boldsymbol{\Sigma}_z - \boldsymbol{\Sigma}_u\boldsymbol{\Sigma}_y^{-1}\boldsymbol{\Sigma}_u^T\right)^{-1}\left(\delta\mu_z - \boldsymbol{\Sigma}_u\boldsymbol{\Sigma}_y^{-1}\delta\mu_y\right) + \frac{N_x-N_y}{2T-N_x-3}\left(\delta\mu_y^T\mathbf{S}_y^{-1}\delta\mu_y\right)^2 \tag{72}$$

Utilizing the fact that $\delta\mu_y$ and $\delta\mu_z - \boldsymbol{\Sigma}_u\boldsymbol{\Sigma}_y^{-1}\delta\mu_y$ are jointly Gaussian and uncorrelated, which means they are independent, we can combine Eqs. (72) and (69) to simplify the expression in Eq. (66) to

$$\begin{aligned}
&\text{cov}\left(\delta\mu_y^T\mathbf{S}_y^{-1}\delta\mu_y, \left(\delta\mu_z - \mathbf{S}_u\mathbf{S}_y^{-1}\delta\mu_y\right)^T\left(\mathbf{S}_z - \mathbf{S}_u\mathbf{S}_y^{-1}\mathbf{S}_u^T\right)^{-1}\left(\delta\mu_z - \mathbf{S}_u\mathbf{S}_y^{-1}\delta\mu_y\right)\right) \\
&= \frac{N_x-N_y}{2T-N_x-3}\left(\mathbb{E}\left(\left(\delta\mu_y^T\mathbf{S}_y^{-1}\delta\mu_y\right)^2\right) - \mathbb{E}\left(\delta\mu_y^T\mathbf{S}_y^{-1}\delta\mu_y\right)^2\right) \\
&= \frac{N_x-N_y}{2T-N_x-3}\text{var}\left(\delta\mu_y^T\mathbf{S}_y^{-1}\delta\mu_y\right) \\
&= \frac{(N_x-N_y)(2T-2)^2\delta\theta^4}{(2T-N_x-3)(2T-N_y-3)^2}\text{var}\left(\hat{I}^y_{g,bc}\right).
\end{aligned} \tag{73}$$

Substituting Eq. (73) into Eq. (65) results in

$$\text{cov}\left(\hat{I}^y_{g,bc}, \delta\hat{I}^{x-y}_{g,bc}\right) = \frac{N_y-N_x}{2T-N_y-3}\text{var}\left(\hat{I}^y_{g,bc}\right) + \frac{N_x-N_y}{2T-N_y-3}\text{var}\left(\hat{I}^y_{g,bc}\right) = 0, \tag{74}$$

which means that information in the smaller population is uncorrelated to the information gain obtained from non-shared neurons. As a consequence,

$$\text{cov}\left(\hat{I}^x_{g,bc}, \hat{I}^y_{g,bc}\right) = \text{var}\left(\hat{I}^y_{g,bc}\right). \tag{75}$$

Note the this only holds for the bias-corrected information estimates. For the naïve estimates, a similar derivation shows that $\hat{I}^y_{g,nv}$ and $\delta\hat{I}^{x-y}_{g,nv}$ are correlated.

Supplementary Note 2. Information scaling models

We assume that information in the recorded population is limited by the presence of information-limiting correlations [1]. In this case, the noise covariance matrix $\mathbf{\Sigma}_N$ for a population of N neurons decomposes into

$$\mathbf{\Sigma}_N = \mathbf{\Sigma}_{0,N} + \frac{1}{I_\infty}\mathbf{f}'_N \mathbf{f}'^T_N, \tag{76}$$

where $\mathbf{\Sigma}_{0,N}$ is the non-limiting covariance component, I_∞ is the asymptotic information, and $\mathbf{f}'_N$ is the derivative of the mean population activity. All of these quantities depend on the stimulus, θ, but we will keep this dependency implicit for notational convenience. In the $N \to \infty$ limit, only the second component limits information, while the information associated with $\mathbf{\Sigma}_{0,N}$ grows without bounds.

To see how information grows in the presence of information-limiting correlations, note that the Sherman-Morrison formula allows us to express $\mathbf{\Sigma}_N^{-1}$ by

$$\mathbf{\Sigma}_N^{-1} = \mathbf{\Sigma}_{0,N}^{-1} - \frac{\mathbf{\Sigma}_{0,N}^{-1}\mathbf{f}'_N \mathbf{f}'^T_N \mathbf{\Sigma}_{0,N}^{-1}}{I_\infty + \mathbf{f}'^T_N \mathbf{\Sigma}_{0,N}^{-1}\mathbf{f}'_N} \tag{77}$$

Let us denote the linear Fisher information associated with the non-limiting component by $I_{0,N} = \mathbf{f}'^T_N \mathbf{\Sigma}_{0,N}^{-1}\mathbf{f}'_N$. Then, after some algebra, the total Fisher information is given by

$$I_N = \mathbf{f}'^T_N \mathbf{\Sigma}_N^{-1}\mathbf{f}'_N = \mathbf{f}'^T_N \mathbf{\Sigma}_{0,N}^{-1}\mathbf{f}'_N - \frac{\mathbf{f}'^T_N \mathbf{\Sigma}_{0,N}^{-1}\mathbf{f}'_N \mathbf{f}'^T_N \mathbf{\Sigma}_{0,N}^{-1}\mathbf{f}'_N}{I_\infty + \mathbf{f}'^T_N \mathbf{\Sigma}_{0,N}^{-1}\mathbf{f}'_N} = \frac{1}{\frac{1}{I_{0,N}} + \frac{1}{I_\infty}}, \tag{78}$$

or, equally, $I_N^{-1} = I_{0,N}^{-1} + I_\infty^{-1}$. This result forms the core of our information scaling models. For the remainder of this section we will discuss how we would expect information $\mathbf{I}_{0,N}$ in the non-limiting component to scale, and the impact of measurement noise on overall information scaling.

2.1 Linear non-limiting information scaling for large N

To characterize the scaling of $I_{0,N}$ with N, let us use the spectral decomposition

$$\mathbf{\Sigma}_{0,N} = \sum_{n=1}^{N} \sigma_{N,n}^2 \mathbf{z}_{N,n}\mathbf{z}_{N,n}^T, \tag{79}$$

with variances $\sigma_{N,1}^2, \dots, \sigma_{N,N}^2$ and principal directions $\mathbf{z}_{N,1}, \dots, \mathbf{z}_{N,N}$. Then, $I_{0,N}$ is given by

$$I_{0,N} = \sum_{n=1}^{N} \frac{\left(\mathbf{f}'^T_N \mathbf{z}_{N,n}\right)^2}{\sigma_{N,n}^2} = \|\mathbf{f}'_N\|^2 \sum_{n=1}^{N} \frac{\cos^2\left(\alpha_{N,n}\right)}{\sigma_{N,n}^2}, \tag{80}$$

where $\alpha_{N,n}$ is the angle between $\mathbf{f}'_N$ and $\mathbf{z}_n$.

To see how $I_{0,N}$ scales with N, let us assume that the $\alpha_{N,n}$'s are independent of the $\sigma_{N,n}^2$'s. Furthermore, $f'_{N,n}$ (that is, the nth component of $\mathbf{f}'_N$) is $\mathcal{O}(1)$, such that $\|\mathbf{f}'_N\|^2$ will be $\mathcal{O}(N)$. In addition, geometry requires that $\sum_{n=1}^N \cos^2\left(\alpha_{N,n}\right) = 1$, such that each $\cos^2\left(\alpha_{N_n}\right)$ is $\mathcal{O}(1/N)$ [1]. Together, this yields

$$I_{0,N} \propto N \sum_{n=1}^{N} \frac{1}{N}\frac{1}{\sigma_{N,n}^2} = \sum_{n=1}^{N} \frac{1}{\sigma_{N,n}^2}. \tag{81}$$

Therefore, under these assumptions, the scaling of $I_{0,N}$ only depends on the scaling of the eigenvalue spectrum $\{\sigma_{N,1}^2, \dots, \sigma_{N,N}^2\}$ of $\mathbf{\Sigma}_{0,N}$.

For the following, we will assume that each neuron in the population features some small amount of "private" noise that is not correlated with the variability of other neurons. This private noise introduces a lower bound,

σ_0^2, on the variances, that is $\sigma_{N,n}^2 \geq \sigma_0^2$ for all n. Together with the above expression, this allows us to derive a lower bound on the scaling of non-limiting information. In particular, by Jensen's inequality

$$I_{0,N} \propto N \left(\frac{1}{N} \sum_{n=1}^{N} \frac{1}{\sigma_{N,n}^2} \right) \geq N \frac{1}{\frac{1}{N} \sum_{n=1}^{N} \sigma_{N,n}^2} \propto N. \tag{82}$$

The second-to-last expression contains the average variance, which is lower-bounded by σ_0^2 and of order one. Therefore, the scaling of $I_{0,N}$ is at least $\mathcal{O}(N)$.

To gain further insight into the scaling of $I_{0,N}$, assume a sequence of non-limiting covariance matrices $\mathbf{\Sigma}_{0,M}, \mathbf{\Sigma}_{0,M-1}, \mathbf{\Sigma}_{0,M-2}, \dots$, starting with some large population with M neurons. Each consecutive matrix $\mathbf{\Sigma}_{0,N-1}$ is constructed from the next-larger matrix $\mathbf{\Sigma}_{0,N}$ by removing a single neuron, such that they share all entries except for one row and column associated with that neuron. If we order their eigenvalues according to $\sigma_{N,1}^2 \geq \sigma_{N,2} \geq \dots \sigma_{N,N}^2$ and $\sigma_{N-1,1}^2 \geq \sigma_{N-1,2}^2 \geq \dots \geq \sigma_{N-1,N-1}^2$, it is known that these eigenvalues obey the interleaved ordering

$$\sigma_{N,1}^2 \geq \sigma_{N-1,1}^2 \geq \sigma_{N,2}^2 \geq \sigma_{N-1,2}^2 \geq \dots \sigma_{N-1,N-1}^2 \geq \sigma_{N,N}^2. \tag{83}$$

Using $I_{0,N} \propto \sum_{n=1}^{N} \sigma_{N,n}^{-2}$, the information increase when moving from $N-1$ to N neurons becomes

$$I_{0,N} - I_{0,N-1} \propto \sum_{n=1}^{N-1} \left(\frac{1}{\sigma_{N,n}^2} - \frac{1}{\sigma_{N-1,n}^2} \right) + \frac{1}{\sigma_{N,N}^2}. \tag{84}$$

This information increase is $\mathcal{O}(1)$ if both terms on the left-hand side are $\mathcal{O}(1)$.

The second term is $\mathcal{O}(1)$ if there exists some positive constant C such that, for all N above some N_0, $\sigma_{N,N}^{-2} \leq C$. As $\sigma_{N,N}^2$ is always the smallest eigenvalue of the covariance matrix, this implies that $\mathcal{O}(1)$ can be guaranteed as long as $\sigma_{N,N}^2$ remains positive with increasing N, which is satisfied by our previous assumption that each neuron has some private noise. If it instead would go to zero, we would have $\lim_{N \to M} \sigma_{N,N}^{-2} = 0$, violating the requirement.

For the first term we observe that the hierarchical eigenvalue relationship of nested matrices implies that $\sigma_{N,n}^{-2} \leq \sigma_{N-1,n}^{-2}$ for all $n = 1, \dots, N-1$. This implies that every element in the sum is negative. However, the information increase $I_{0,N} - I_{0,N-1}$ cannot be negative. Therefore, the second term on the left-hand side has to be at least as large as the negative first term (i.e., the sum), that is

$$\frac{1}{\sigma_{N,N}^2} \geq - \sum_{n=1}^{N} \left(\frac{1}{\sigma_{N,n}^2} - \frac{1}{\sigma_{N-1,n}^2} \right). \tag{85}$$

As $\sigma_{N,N}^{-2}$ is $\mathcal{O}(1)$, the sum cannot be larger than $\mathcal{O}(1)$. Overall, as long as none of the variances become zero with increasing N, the increase in $I_{0,N}$ will be $\mathcal{O}(1)$, which implies that $I_{0,N}$ scales with $\mathcal{O}(N)$.

2.2 Models for $I_{0,N}$

The above argument shows that, under rather general conditions, $I_{0,N}$ can be expected to scale with $\mathcal{O}(N)$. However, it does not tell us about how $I_{0,N}$ behaves for small N, which depends on the details of the structure of $\mathbf{\Sigma}_{N,0}$.

To describe the details of this structure, we compared two models for $I_{0,N}$. The first, called the *lim* model, directly follows the scaling results and assumes that $I_{0,N} = cN$ with some parameter c that is independent of N. The second model, called the *lim-exp* model, allows the non-limiting information to initially grow supralinearly before converging to a linear growth. We derived this model by integrating $c\left(1 - e^{-N/\tau}\right)$ from zero to N, resulting in

$$I_{0,N} = c \left(N + \tau \left(e^{-\frac{N}{\tau}} - 1 \right) \right), \tag{86}$$

with the additional parameter τ that controls the extent of the initial supralinearity (in units of N). We have chosen this particular model, as it turns out easier to fit than alternative models (such as, for example, integrating

a re-scaled logistic sigmoid over the positive half-line) that provide qualitatively similar qualitative $I_{0,N}$ scaling. This model approaches $I_{0,N} = cN$ in the $\tau \to 0$ limit. Model comparison revealed the *lim* model to significantly outperform the *lim-exp* model (Supplementary Figure 6), such that we focused on the *lim* model in the main text.

2.3 Impact of measurement noise

Our recordings of neural activity might be noisy, introducing additional variability into our estimates of $\mathbf{\Sigma}_N$ and $\mathbf{f}'_N$. To estimate the effect of such measurement noise, we assume it to be of equal magnitude and independent across neurons, such that it adds an additional diagonal term to the covariance decomposition,

$$\mathbf{\Sigma}_N = \mathbf{\Sigma}_{0,N} + \frac{1}{I_\infty}\mathbf{f}'_N \mathbf{f}'^T_N + \sigma^2_{rec}\mathbf{I}, \tag{87}$$

where σ^2_{rec} denotes the variance of the measurement noise. We don't assume it to impact differential correlations, as those limit information *in the brain*, rather than our measurement thereof.

Following the same derivation as in the beginning of this section, the information in a population of N neurons becomes

$$I_N = \frac{1}{\frac{1}{I_{0,rec,N}} + \frac{1}{I_\infty}}, \tag{88}$$

where

$$I_{0,rec,N} = \mathbf{f}'^T_N \left(\mathbf{\Sigma}_{0,N} + \sigma^2_{rec}\mathbf{I}\right)^{-1} \mathbf{f}'_N, \tag{89}$$

is the non-limiting information, including measurement noise. We can, as before, use the spectral decomposition $\mathbf{\Sigma}_{0,N} = \sum_{n=1}^N \sigma^2_{N,n}\mathbf{z}_n\mathbf{z}_n^T$ and observe that $\mathbf{I} = \sum_{n=1}^N \mathbf{z}_n\mathbf{z}_n^T$, resulting in

$$\mathbf{\Sigma}_{0,N} + \sigma^2_{rec}\mathbf{I} = \sum_{n=1}^N \left(\sigma^2_{N,n} + \sigma^2_{rec}\right)\mathbf{z}_n\mathbf{z}_n^T. \tag{90}$$

This shows that measurement noise increases all eigenvalues of $\mathbf{\Sigma}_{0,N}$ by the same magnitude.

This has several consequences. First, the added variance baseline results in $I_{0,rec,N}$ to grow more slowly with N than $I_{0,N}$. Second, this baseline causes in the eigenvalues of $\mathbf{\Sigma}_{0,N} + \sigma^2_{rec}\mathbf{I}$ to be more similar to each other than those of $\mathbf{\Sigma}_{0,N}$ alone. As a consequence, the growth of $I_{0,rec,N} \propto \sum_{n=1}^N \left(\sigma^2_{N,n} + \sigma^2_{rec}\right)^{-1}$ with N is more linear than that of $I_{0,N} \propto \sum_{n=1}^N \sigma^{-2}_{N,n}$. This might make $I_{0,rec,N} = c_{rec}N$ a good model of non-limiting information growth, even if $I_{0,N} = cN$ is not. Third, as the measurement noise impacts only $I_{0,rec,N}$ but not I_∞, measurement noise only impacts our estimates of c but not of I_∞. Fourth, measurement noise will lower our estimates of c, and therefore increase our estimates of $N_a = a/(1-a)I_\infty/c$, which is the population size at which a fraction a of the asymptotic information I_∞ is reached.

2.4 Impact of eye movements

To estimate the impact of eye movements that might occur between trials, we assume that the only impact that they might have had was to have the stimulus appear outside some neurons' receptive field in some trials. We assumed that, under those circumstances, the neuron's activity would be set to zero. For tractability, our model does not consider details of the spatial structure of receptive fields. This leads to a simple model in which we assume one indicator variable z_t per trial t that is $z_t = 0$ if the stimulus appears in all measured neurons' receptive fields in that trial, and $z_t = 1$ if it might fall outside of some receptive fields. We furthermore introduce the indicator variable z_{nt} for neuron n in trial t that is $z_{nt} = 0$ if the stimulus falls into neuron n's receptive field in trial t, and $z_{nt} = 1$ if it might fall outside of it. We assume that $p(z_t = 1) = p_t$ and $p(z_{nt} = 1) = p_n$, independent across trials and neurons. Furthermore, p_n is the same across all neurons, and thus a scalar. Neuron n's activity in trial t is set to zero only if $z_t = 1$ *and* $z_{nt} = 1$. Formally, if $r_{nt,ori}$ is the neuron's unperturbed (original) spike count in that trial, its perturbed one becomes

$$r_{nt} = (1 - z_t z_{nt})r_{nt,ori}. \tag{91}$$

For simplicity we here assumed z_t and z_{nt} to be independent, and are zeroing neurons only if $z_t z_{nt} = 1$, jointly. For $z_t = 0$, this occurs with probability $p(z_t z_{nt} = 1|z_t = 0) = 0$, that is, never. For $z_t = 1$, it occurs with probability $p(z_t z_{nt} = 1|z_t = 1) = p_n$.

We could have equivalently fixed $z_{nt} = 0$ for all trials in which $z_t = 0$, defined $p(z_{nt} = 1|z_t = 1) = p_n$, and set $r_{nt} = (1 - z_{nt})r_{nt,ori}$. This choice leads to $p(z_{nt} = 1|z_t = 0) = 0$ and $p(z_{nt} = 1|z_t = 1) = p_n$, illustrating that it yields the same result as our original assumptions. In either formulation, z_t mimics global fluctuations, whereas z_{nt} mimics fluctuations that are private to individual neurons.

2.4.1 Impact on population activity moments

As linear Fisher information only depends on $\mathbf{f}'$ and $\mathbf{\Sigma}$ we here assess the impact on occasionally zeroing out neurons on these moments. To do so, we denote $\mathbf{f}_{ori}$, $\mathbf{f}'_{ori}$, and $\mathbf{\Sigma}_{ori}$ as the unperturbed moments, and $\mathbf{f}$, $\mathbf{f}'$, and $\mathbf{\Sigma}$ as the same moments after perturbation. To find the perturbed mean, observe that

$$f_n = \langle r_{nt} \rangle = \langle (1 - z_t z_{nt}) r_{nt,ori} \rangle = (1 - p_t p_n) \langle r_{nt,ori} \rangle = (1 - p_t p_n) f_{n,ori}, \tag{92}$$

such that $\mathbf{f} = (1 - p_t p_n)\mathbf{f}_{ori}$. As $\mathbf{f}'$ is the difference between two $\mathbf{f}$'s, we furthermore have $\mathbf{f}' = (1 - p_t p_n)\mathbf{f}'_{ori}$.

The perturbed covariance follows a similar derivation, for $n \neq m$,

$$\begin{aligned}
\Sigma_{nm} &= \langle r_{nt} r_{mt} \rangle - \langle r_{nt} \rangle \langle r_{mt} \rangle \\
&= \langle (1 - z_t z_{nt})(1 - z_t z_{mt}) r_{nt,ori} r_{mt,ori} \rangle - \langle (1 - z_t z_{nt}) r_{nt,ori} \rangle \langle (1 - z_t z_{mt}) r_{mt,ori} \rangle \\
&= \langle (1 - z_t (z_{nt} + z_{mt} - z_{nt} z_{mt})) r_{nt,ori} r_{mt,ori} \rangle - (1 - p_t p_n)^2 f_{n,ori} f_{m,ori} \\
&= (1 - p_t p_n (2 - p_n)) \langle r_{nt,ori} r_{mt,ori} \rangle - (1 - p_t p_n)^2 f_{n,ori} f_{m,ori} \\
&= (1 - p_t p_n (2 - p_n)) (\langle r_{nt,ori} r_{mt,ori} \rangle - \langle r_{nt,ori} \rangle \langle r_{mt,ori} \rangle) + p_t (1 - p_t) p_n^2 f_{n,ori} f_{m,ori} \\
&= (a^2 + b^2) \Sigma_{nm,ori} + b^2 f_{n,ori} f_{m,ori},
\end{aligned} \tag{93}$$

where we have defined

$$a = 1 - p_t p_n, \qquad b = p_n \sqrt{p_t (1 - p_t)}. \tag{94}$$

A similar derivation for $n = m$ results in

$$\Sigma_{nn} = (a^2 + b^2) \Sigma_{nn,ori} + b^2 f_{n,ori}^2 + p_t p_n (1 - p_n) \left(\Sigma_{nn,ori} + f_{n,ori}^2 \right). \tag{95}$$

Together, this yields

$$\Sigma_{nm} = (a^2 + b^2) \Sigma_{nm,ori} + b^2 f_{n,ori} f_{m,ori} + \delta_{nm} p_t p_n (1 - p_n) \left(\Sigma_{nn,ori} + f_{n,ori}^2 \right), \tag{96}$$

where $\delta_{nm} = 1$ if $n = m$, and $\delta_{nm} = 0$ otherwise.

Overall, this results in

$$\mathbf{\Sigma} = \left(a^2 + b^2 \right) \mathbf{\Sigma}_{ori} + b^2 \mathbf{f}_{ori} \mathbf{f}_{ori}^T + p_t p_n (1 - p_n) \mathbf{S}, \tag{97}$$

where $\mathbf{S}$ is a diagonal matrix with $\Sigma_{nn,ori} + f_{n,ori}^2$ as the nth element of its diagonal. Therefore, the perturbed covariance $\mathbf{\Sigma}$ is a scaled-down version (as $a^2 + b^2 \leq 1$) of the unperturbed covariance $\mathbf{\Sigma}_{ori}$, with the scaling factor $a^2 + b^2$ decreasing monotonically in both p_n and p_t. Zeroing out neurons results in two additional perturbations. First, it causes the addition of the rank-one matrix $\mathbf{f}_{ori}\mathbf{f}_{ori}^T$ whose magnitude increases with p_n and the variance of z_t, $\text{var}(z_t) = p_t(1 - p_t)$, which is largest for $p_t = 1/2$. Second, it adds a diagonal component whose magnitude increases with p_t and the variance of z_{nt}, $\text{var}(z_{nt}) = p_n(1 - p_n)$.

Both $\mathbf{f}$ and $\mathbf{\Sigma}$ are computed conditional on a specific stimulus. The $\mathbf{\Sigma}$ we used to compute linear Fisher information is the average across the two considered stimuli. Then, the perturbed $\mathbf{\Sigma}$ becomes a linear combination of the average $\mathbf{\Sigma}_{ori}$, $\mathbf{f}_{ori}\mathbf{f}_{ori}^T$, and $\mathbf{S}$, for both stimuli.

2.4.2 Impact on information scaling

Let us consider the impact of the above perturbations on how information scales with population size, and its consequences for the estimated scaling parameters c and I_∞. If we ignore the rank-one and diagonal additions to the covariance matrix, the overall Fisher information for each N is re-scaled by

$$I_N \approx \frac{(1 - p_n p_t)^2}{a^2 + b^2} I_{N,ori}, \tag{98}$$

with a scaling factor that is close-to one for small p_n and p_t, and shrinks monotonically in p_n. Furthermore, it is smallest for interim values of p_t, but becomes one for $p_t = 0$ and $p_t = 1$. Based on the expression of our information scaling model, Eq. (78), as $I_{N,ori}^{-1} = c_{ori}^{-1} N^{-1} + I_{\infty,ori}^{-1}$, estimates of both c and I_∞ will be down-scaled by the same factor.

Adding a diagonal term to $\mathbf{\Sigma}$ has an analogous effect as measurement noise, as discussed in the previous section: it causes information to grow more slowly with N, thus lowering c further, but leaves I_∞ unperturbed.

The impact of the rank-one component $\mathbf{f}_{ori}\mathbf{f}_{ori}^T$ depends on the alignment between $\mathbf{f}_{ori}$ and $\mathbf{f}'_{ori}$. If they are perfectly aligned, that is, if $\mathbf{f}_{ori} \propto \mathbf{f}'_{ori}$, this component introduces differential correlations, thus lowering asymptotic information I_∞. If alignment is only partial, then this component does not limit information [1], but might still lower c. Non-alignment is likely if a change in the stimulus results in a simple shift in the population activity pattern, as is the case in our experiments. Perfect alignment could, for example, occur, if the stimulus modulates the gain of population activity, making the change in population activity due to a change in stimulus well-aligned with the average population activity. This might, for example, occur in contrast discrimination tasks, if contrast modulates the population activity gain.

In summary, zeroing out some neurons in a fraction of trials results in lowering our estimate of c, depending on a complex interplay of p_t and p_n. If $\mathbf{f}_{ori}$ is not perfectly aligned to $\mathbf{f}'_{ori}$, then I_∞ is down-weighted by $(1 - p_n p_t)^2/(a^2 + b^2)$. In case of perfect alignment, the asymptotic information estimate is suppressed further. We confirmed these effects in simulations, as shown in Supplementary Figure 14.

Supplementary Note 3. Estimating the information scaling moments from neural data

Here, we fix the discrimination (i.e., the pair of drift directions, θ_1 and θ_2) and discuss how we estimate the moments of Fisher information for different population sizes. To do so, we assume a large population with M neurons of which we subsample N neurons, and where $N \ll M$. Rather than focusing on the moments of the Fisher information I_n for population size $n \leq N$, we will instead focus on the moments of the Fisher information increase, $\Delta I_n = I_n - I_{n-1}$ (with $I_0 = 0$), when increasing the population size from $n-1$ to n neurons, for reasons that become apparent later. Our aim is to estimate the mean, $\mathbb{E}(\Delta I_n)$, the variance, var (ΔI_n), and the covariance, cov $(\Delta I_n, \Delta I_m)$, for different population sizes n and m.

3.1 Generative model and desired moments

To describe the stochasticity of ΔI_n, we assume the following generative process. Assume that neurons in the large population have indices 1 to M, and that we uniformly draw a subset of N different neurons with indices $i_1, i_2, \dots, i_N$, denoted $i_{1:N}$. This subpopulation has moments $\mathbf{f}'_{i_{1:N}}$ and $\mathbf{\Sigma}_{i_{1:N}}$, that in turn can be used to compute its associated Fisher information. However, we do not directly observe these moments, but instead record the population activity across T trials for each stimulus, θ_1 and θ_2, from which we compute the empirical moments $\gamma_{i_{1:N}}$ and $\mathbf{\Omega}_{i_{1:N}}$. These empirical moments are in turn used to compute the Fisher information increases $\Delta \hat{I}_{1:N}$, using the bias-corrected estimates discussed further above. In summary, the generative process follows the Markov chain

$$i_{1:N} \rightarrow \mathbf{f}'_{i_{1:N}}, \mathbf{\Sigma}_{i_{1:N}} \rightarrow \gamma_{i_{1:N}}, \mathbf{\Omega}_{i_{1:N}} \rightarrow \Delta \hat{I}_{1:N}. \tag{99}$$

In this Markov chain, the first and last transition are deterministic, and the center transition is stochastic. Therefore, we can write the generative model as

$$p\left(\Delta \hat{I}_{1:N}\right) = \sum_{i_{1:N}} p\left(\Delta \hat{I}_{1:N}\left(\gamma_{i_{1:N}}, \mathbf{\Omega}_{i_{1:N}}\right) | i_{1:N}\right) p\left(i_{1:N}\right), \tag{100}$$

where the Fisher information increases are a deterministic function of the empirical moments, and the sum is over different subpopulations drawn from the larger population. We assume these draws to be uniform, that is $p\left(i_{1:N}\right) \propto 1$.

To find the moments of $\Delta \hat{I}_n$, we use iterated expectation, variance, and covariance, which, for a Markov chain $Z \rightarrow X_1, X_2$ is given by

$$\mathbb{E}_{X_1}\left(X_1\right) = \mathbb{E}_Z\left(\mathbb{E}_{X_1|Z}\left(X_1\right)\right), \tag{101}$$

$$\text{var}_{X_1}\left(X_1\right) = \mathbb{E}_Z\left(\text{var}_{X_1|Z}\left(X_1\right)\right) + \text{var}_Z\left(\mathbb{E}_{X_1|Z}\left(X_1\right)\right), \tag{102}$$

$$\text{cov}_{X_1,X_2}\left(X_1, X_2\right) = \mathbb{E}_Z\left(\text{cov}_{X_1,X_2|Z}\left(X_1, X_2\right)\right) + \text{cov}_Z\left(\mathbb{E}_{X_1|Z}\left(X_1\right), \mathbb{E}_{X_2|Z}\left(X_2\right)\right). \tag{103}$$

Applied to our generative model, that yields the decompositions

$$\mathbb{E}_{\Delta \hat{I}_n}\left(\Delta \hat{I}_n\right) = \mathbb{E}_{i_{1:N}}\left(\mathbb{E}_{\Delta \hat{I}_n|i_{1:N}}\left(\Delta \hat{I}_n\right)\right), \tag{104}$$

$$\text{var}_{\Delta \hat{I}_n}\left(\Delta \hat{I}_n\right) = \mathbb{E}_{i_{1:N}}\left(\text{var}_{\Delta \hat{I}_n|i_{1:N}}\left(\Delta \hat{I}_n\right)\right) + \text{var}_{i_{1:N}}\left(\mathbb{E}_{\Delta \hat{I}_n|i_{1:N}}\left(\Delta \hat{I}_n\right)\right), \tag{105}$$

$$\text{cov}_{\Delta \hat{I}_n, \Delta \hat{I}_m}\left(\Delta \hat{I}_n, \Delta \hat{I}_m\right) = \mathbb{E}_{i_{1:N}}\left(\text{cov}_{\Delta \hat{I}_n, \Delta \hat{I}_m|i_{1:N}}\left(\Delta \hat{I}_n, \Delta \hat{I}_m\right)\right) + \text{cov}_{i_{1:n}}\left(\mathbb{E}_{\Delta \hat{I}_n|i_{1:N}}\left(\Delta \hat{I}_n\right), \mathbb{E}_{\Delta \hat{I}_m|i_{1:N}}\left(\Delta \hat{I}_m\right)\right), \tag{106}$$

where both variance and covariance are decomposed into (i) the (co)variance of the information increase for a fixed subpopulation $i_{1:N}$, averaged across different subpopulations, and (ii) how the average information increase for a fixed subpopulation (co)varies across different subpopulations.

Our data does not allow us to directly estimate these moments for two reasons. First, we don't observe the larger population, and so can't use it to draw different subpopulations from this larger population. We will

address how we handle this limitation in the next subsection. Second, we only observe a single set of empirical moments, μ and S, for the subpopulation that we record from. We will address how we handle this limitation in the remaining subsections.

3.2 Simulating samples from a large, unobserved population

Our generative model assumes that we are subsampling N neurons from a large neural populations of M neurons. Our data, in contrast, are population recordings from a single neural population with N neurons. To use these recordings to simulate sampling from various subpopulations of the larger population, we assume these subpopulations to be statistically similar to the recorded population. That is, the different sampled subpopulations will contain neurons with similar activity statistics as the recorded population. Thus, each sampled subpopulation will contain all neurons from the recorded population, but in a different order for each sampled subpopulation. We will simulate this by introducing a new index set $j_{1:N} = j_1, j_2, \dots, j_N$ that, for each sampled subpopulation $i_{1:N}$, contains a random order of the indices $1, \dots, N$ of neurons in the recorded population. With this, all of the above moments across $i_{1:N}$ will become moments across $j_{1:N}$, while taking into account that the recorded subpopulation is used as a proxy for sampling different subpopulations from a larger populations. We will describe the consequences of this for each of the moments separately.

3.3 Estimating the mean

The desired mean of the information increase $\Delta\hat{I}_n$ is, by Eq. (104) the average information increase for a particular set of empirical moments, $\gamma_{i_{1:N}}$ and $\mathbf{\Omega}_{i_{1:N}}$, for a particular subpopulation $i_{1:N}$, averaged across different subpopulations. We deal with not being able to sample different subpopulations by replacing $i_{1:N}$ by a randomly ordered recorded population $j_{1:N}$. Furthermore, we cannot draw different empirical moments, $\gamma_{i_{1:N}}$ and $\mathbf{\Omega}_{i_{1:N}}$ for a given subpopulation, as would be required to compute $\mathbb{E}_{\Delta\hat{I}|i_{1:N}}\left(\Delta\hat{I}_n\right)$. We will replace this expectation with our best estimate thereof, which is the Fisher information increase estimate based on the bias-correctet Fisher information, estimated from the empirical moments of the recorded population, μ and S. Overall, this leads to the approximate estimate,

$$\mathbb{E}_{i_{1:N}}\left(\mathbb{E}_{\Delta\hat{I}_n|i_{1:N}}\left(\Delta\hat{I}_n\right)\right) \approx \mathbb{E}_{j_{1:N}}\left(\Delta\hat{I}_n\left(\mu_{j_{1:N}}, \mathbf{S}_{j_{1:N}}\right)\right), \tag{107}$$

where $\mu_{j_{1:N}}$ and $\mathbf{S}_{j_{1:N}}$ denote the empirical moments with neurons ordered according to $j_{1:N}$. As our Fisher information estimate is unbiased, the above estimate will be unbiased as well. In practice, we approximate the expectation over $j_{1:N}$ by 10000 random ordering.

3.4 Estimating the variance

The variance, Eq. (105), is decomposed into two terms. The first, $\mathbb{E}_{i_{1:N}}\left(\text{var}_{\Delta\hat{I}_n|i_{1:N}}\left(\Delta\hat{I}_n\right)\right)$, is the variance of the Fisher information increase for a fixed subpopulation, averaged across many subpopulations. This term captures the uncertainty in $\Delta\hat{I}_n$ due to using the empirical moments to estimate it. The second term, given $\text{var}_{i_{1:N}}\left(\mathbb{E}_{\Delta\hat{I}_n|i_{1:N}}\left(\hat{I}_n\right)\right)$, captures how the average Fisher information increase for a given subpopulation varies across different subpopulations. Our data doesn't allow us to compute either of these terms directly. However, it turns out that they are both well-approximated by how the Fisher information increase estimated from the empirical moments, μ and S, varies across different population orders, $j_{1:N}$, that is

$$\mathbb{E}_{i_{1:N}}\left(\text{var}_{\Delta\hat{I}_n|i_{1:N}}\left(\Delta\hat{I}_n\right)\right) + \text{var}_{i_{1:N}}\left(\mathbb{E}_{\Delta\hat{I}_n|i_{1:N}}\left(\Delta\hat{I}_n\right)\right) \approx \text{var}_{j_{1:N}}\left(\Delta\hat{I}_n\left(\mu_{j_{1:N}}, \mathbf{S}_{j_{1:N}}\right)\right). \tag{108}$$

To understand why this approximation works, we need to consider two components that contribute to the empirical moments of the recorded neurons. The first is that, for each neuron and each neuron pair, these empirical moments are noisy, as they are estimated from a limited number of trials. Thus, we can approximate the effect of using empirical rather than true moments, as captured by the first term in Eq. (105), by computing the

variance across different neurons in the population, as achieved by the variance across different orderings, $j_{1:N}$. The second factor is that different neurons contribute different amounts of information to the population. This comes into play in the second term in Eq. (105), and is again well-approximated by the variance across different orderings, $j_{1:N}$. As it seems paradoxical that the same variance can capture both kinds of effects at the same time, we have demonstrated it in simulations of neural populations, shown in Supplementary Figure 16a.

3.5 Estimating the covariance

As the variance, the covariance, Eq. (106) can be decomposed into two terms that capture different sources of uncertainty. The first term, $\mathbb{E}_{i_{1:N}}\left(\text{cov}_{\Delta\hat{I}_n,\Delta\hat{I}_m|i_{1:N}}\left(\Delta\hat{I}_n,\Delta\hat{I}_m\right)\right)$, captures the uncertainty associated with estimating empirical moments from a limited number of trials. To find this covariance, assume $n \neq m$ and note that,

$$\begin{aligned}\text{cov}\left(\Delta\hat{I}_n,\Delta\hat{I}_m\right) &= \text{cov}\left(\hat{I}_n-\hat{I}_{n-1},\hat{I}_m-\hat{I}_{m-1}\right)\\ &= \text{cov}\left(\hat{I}_n,\hat{I}_m\right)-\text{cov}\left(\hat{I}_n,\hat{I}_{m-1}\right)-\text{cov}\left(\hat{I}_{n-1},\hat{I}_m\right)+\text{cov}\left(\hat{I}_{n-1},\hat{I}_{m-1}\right)\end{aligned} \tag{109}$$

where all covariances are conditional on $i_{1:N}$. Without loss of generality we can assume that $n > m$, and use Eq. (75) from Sec. 1.4 to find

$$\text{cov}\left(\Delta\hat{I}_n,\Delta\hat{I}_m\right) = \text{var}\left(\hat{I}_m\right)-\text{var}\left(\hat{I}_{m-1}\right)-\text{var}\left(\hat{I}_m\right)+\text{var}\left(\hat{I}_{m-1}\right) = 0. \tag{110}$$

This shows, that, conditional on $i_{1:N}$, the information increase estimates are uncorrelated.

The second term, $\text{cov}_{i_{1:N}}\left(\mathbb{E}_{\Delta\hat{I}_n|i_{1:N}}\left(\Delta\hat{I}_n\right),\mathbb{E}_{\Delta\hat{I}_m|i_{1:N}}\left(\Delta\hat{I}_m\right)\right)$, captures how the average Fisher information increase associated with adding the nth neuron correlates with that when adding the mth neuron across different subpopulation samples. On average, these increases will be negatively correlated, for the following reason. The variance of the information estimate $\hat{I}_n = \sum_{k=1}^{n}\Delta\hat{I}_k$ can be decomposed into

$$\text{var}\left(\hat{I}_n\right) = \sum_{k=1}^{n}\left(\text{var}\left(\Delta\hat{I}_k\right)+2\sum_{l=1}^{k-1}\text{cov}\left(\Delta\hat{I}_k,\Delta\hat{I}_l\right)\right), \tag{111}$$

which shows the impact of the individual variances, as well as the covariance between estimates associated with different population sizes. For a population of M neurons, the estimate of total information, $\hat{I}_M$, will be the same, irrespective of how the neurons are ordered within that subset. Therefore, $\text{var}\left(\hat{I}_M\right) = 0$. However, as, by definition, $\text{var}\left(\Delta\hat{I}_n\right) \geq 0$, the above decomposition implies that the covariances need to be on average negative, to ensure that the sum of variances and covariances becomes zero.

The same principle applies if we estimate the variance of $\Delta\hat{I}_n$ by shuffling the order, $j_{1:N}$, of neurons in a smaller, recorded population. If this population has N neurons, then $\text{var}\left(\mathbb{E}_{\hat{I}_N|j_{1:N}}\left(\hat{I}_N\right)\right) = 0$, irrespective of $j_{1:N}$, such that the information increase estimates will be negatively correlated.

Recall that we use population order shuffling as a proxy for repeatedly subsampling N neurons from a larger population of M neurons. The shuffling-induced negative correlations arise from using the same N recorded neurons across all estimates. If we instead subsample a larger population, the different sampled subpopulations are bound to share a smaller number of neurons. For two subpopulations that share no neurons, these estimates would be completely uncorrelated. However, even for $N \ll M$, two random subpopulations of size N are likely to share neurons of the larger population. Indeed, the same intuition underlying the birthday paradox [14] tells us that we are almost guaranteed to find such shared neurons. However, the correlations don't only depend on the presence of shared neurons, but also on how many of them are shared, and the latter will decrease significantly for larger M. To show that this significantly lowers the impact of negative correlations on the total variance, we compare this variance computed with and without accounting for these correlations for different

M's. As Supplementary Figure 16b shows, their impact drops significantly with growing M. Therefore, we will approximate them to be zero, that is

$$\mathrm{cov}_{\Delta\hat{I}_n,\Delta\hat{I}_m}\left(\Delta\hat{I}_n,\Delta\hat{I}_m\right)\approx 0. \tag{112}$$

This results in an overestimate of the variance of the Fisher information estimate, and make our fits less certain, and, as a consequence, more conservative.

Supplementary Note 4. Population activity models

We used two different models to simulate population activity, as described below.

4.1 A Gaussian population activity model with limited information

We used a simple Gaussian activity model to satisfy the assumptions of Gaussianity underlying the generalized linear Fisher information, and to test some of the properties of our estimates. This model violates some properties of neural activity, like non-negativity, but is convenient for our purposes, as it supports fine control over the eigenvalues of $\mathbf{\Sigma}_0$, the alignment of $\mathbf{f}'$ to $\mathbf{\Sigma}_0$, and the asymptotic information, I_∞. For a population size of N neurons, we generated $\mathbf{\Sigma}_0$ by drawing a random orthonormal matrix $\mathbf{Z}_0$ of size $N \times N$ that forms the eigenvectors of $\mathbf{\Sigma}_0$. We parameterized the eigenvalues by $\sigma_{n,0}^2 = \sigma_0^2 + \sigma_b m^{-\beta}$, which together form the diagonal matrix $\mathbf{D}_0 = \operatorname{diag}\left(\sigma_{0,1}^2, \ldots, \sigma_{0,N}^2\right)$. $\mathbf{Z}_0$ and $\mathbf{D}_0$ together specify $\mathbf{\Sigma}_0$ by $\mathbf{\Sigma}_0 = \mathbf{Z}_0 \mathbf{D}_0 \mathbf{Z}_0^T$. For a given $\mathbf{f}'$, the full noise covariance is then given by $\mathbf{\Sigma} = \mathbf{\Sigma}_0 + I_\infty^{-1} \mathbf{f}' \mathbf{f}'^T$.

For Supplementary Figure 7, we drew a random $\mathbf{f}' \sim \mathcal{N}(\mathbf{0}, \mathbf{I})$, and subsequently rescaled the vector such that $\|\mathbf{f}'\| = g$. This makes the alignment of $\mathbf{f}'$ to the eigenvectors of $\mathbf{\Sigma}_0$ roughly uniform on average. For this figure, we use parameters $I_\infty = 20$ (or $I_\infty = \infty$ for the unlimited-information case), $g = 20$, $\sigma_0^2 = 10^{-3}$, $\sigma_b^2 = 1$, and $\beta = 0.1$.

For Supplementary Figure 16, we specified the alignment of $\mathbf{f}'$ to the eigenvectors of $\mathbf{\Sigma}_0$ by $\alpha_n = \sigma_\alpha^2 + \propto e^{-n/\tau_\alpha}$, normalized such that $\sum_n \alpha_n = 1$. This yields $\tilde{\mathbf{f}}' = \sum_n \alpha_n \mathbf{z}_n$ ($\mathbf{z}_n$ is the nth eigenvector of $\mathbf{\Sigma}_0$), and $\mathbf{f}' = \sqrt{g_f N} \tilde{\mathbf{f}}' / \|\tilde{\mathbf{f}}'\|$. The magnitude of $\mathbf{f}'$ here scales with $\sqrt{N}$ to ensure roughly similar information across different N's. The used parameters were $I_\infty = 100$, $g_f = 0.008$, $\sigma_0^2 = 5 \times 10^{-5}$, $\sigma_g^2 = 3$, $\beta = 0.5$, $\sigma_\alpha^2 = 10^{-3}$, and $\tau_\alpha = 30$, which results in population statistics comparable to those shown in Figs. 3 and 8 in the main text.

4.2 A visual hierarchy population activity model

We relied on [15] for a more realistic model of V1 population activity that is driven by pixel-level inputs. Details of this model can be found in [15]. Briefly, a population of N neurons responded to a $P \times P$ pixelated images $\mathbf{J}$ of an oriented Gabor. The nth neuron's linear filter $\mathbf{F}_n$ was for each (x, y) pixel determined by

$$c e^{-\frac{(x^2+y^2)}{2\sigma^2}} \cos\left(\frac{2\pi x}{\lambda} \cos(\theta_n) + \frac{2\pi y}{\lambda} \sin(\theta_n) + \phi\right), \tag{113}$$

where c is the Michelson contrast, θ_n determines the neuron's tuning, σ^2 determines the size of the exponential envelope, and λ and ϕ are the Gabor's frequency and phase, respectively. The filter was computed by the above function for each (x, y) and then standardized to have mean zero and unit variance across all (x, y). Image templates, $\mathbf{J}(\theta)$, in response to stimulus θ were generated equally, with θ_n replaced by the template's orientation, θ. Each neuron's gain, a_n, was drawn from a log-normal distribution with unit mean and variance σ_a^2, and then multiplied by the overall gain, g.

Neural population activity is assumed to arise from the image template with Gaussian pixel noise (zero mean, variance σ_0^2), followed by application of the per-neuron linear filters, $\mathbf{F}_n$, multiplied by their gain a_n, and a Poisson step. For Supplementary Figure 7, we estimated information from a set of trials, in each of which neural activity was generated from a different pixel noise instantiation. For Supplementary Figure 10, we skipped the Poisson step, as it introduced additional noise and was not required for the point we were trying to make. Instead, we estimated Fisher information from approximations to the neural mean responses and their covariance matrix, following [15]. We computed the mean response of neuron n to image $\mathbf{J}$ by $f_n(\theta) = \left[a_n \sum_{xy} F_{n,xy} J_{xy}(\theta)\right]_+$, where $[\cdot]_+$ is the threshold-linear function that sets negative values to zero. The population noise covariance was computed by

$$\mathbf{\Sigma}(\theta) = \sigma_0^2 \left(\mathbf{a}\mathbf{a}^T\right) \otimes \left[\mathbf{F}^T \mathbf{F}\right]_+ + \operatorname{diag}\left(\mathbf{a} \otimes \mathbf{f}(\theta)\right), \tag{114}$$

where $\otimes$ denotes the (element-wise) Hadamard product, $\mathbf{a} = (a_1, \ldots, a_N)^T$ is the column vector of per-neuron gains, $\mathbf{F}$ is the $P^2 \times N$ filter matrix with per-neuron filters unrolled as vectors along its columns, and $\mathbf{f}(\theta)$ is the mean population activity in response to stimulus θ. The information was computed from $\mathbf{\Sigma}(\theta)$ and $\mathbf{f}(\theta)$.

For Figs. 7, 14, and 15 we used the parameters $\sigma = P/5$, $\lambda = P/1.5$, $\phi = 0$, $c = 1$, $g = 20$, and $\sigma_a = \sqrt{2}$, as in [15], and additionally different N's for different figures, $P = 32$ and $\sigma_0 = 0.25$. To simulate infinite information, we removed pixel noise by setting $\sigma_0 = 0$. For Supplementary Figure 10, we used the same parameters except $N = 1000$, $g = 10$, and $\sigma_0 = 0.11$, to achieve the desired level of information, and approximate information saturation within the simulated population size. In all simulations, neural tuning, θ_n, was uniformly distributed over $[-\pi, \pi]$, and pixels (x, y) were uniformly distributed over locations $[-(P-1)/2, (P-1)/2]$ in both dimensions.

5 Supplementary Tables

		Session						
Mouse	Contrast	A	B	C	D	E	F	G
1	10%	2.03%	2.65%					
2	10%	2.03%	3.11%					
3	10%	3.21%	2.44%	5.49%	6.23%	3.55%		
4	10%	5.19%	4.70%	4.46%	5.13%	3.66%	2.30%	5.38%
5	10%	4.13%	2.29%	2.84%	1.00%			
	25%	3.36%	1.72%	0.52%	0.33%			
6	10%	3.57%	2.34%	2.37%				
	25%	2.30%	1.34%	4.44%				

Supplementary Table 1: Percentage of neurons that show significant adaptation at the $p = 0.05$ level, for all sessions/mice. We assessed adaptation by asking if the response of each neuron to a drifting stimulus was significantly modulated by the drift direction of the preceding stimulus. We did so by asking if we could reject a non-adaptive model when comparing it to an adaptive model. The non-adaptive model fit neural responses r_i across trials $i = 1, 2, \dots$ by linear regression, using the model

$$r_i \sim \sum_{j=1}^{8} 1_{x_i=\theta_j} \beta_j, \tag{115}$$

where x_i is the stimulus' drift direction in trial i, θ_j is the j's of the eight drift direction used in our experiments, and 1_a is the indicator function that results in $1_a = 1$ if a is true, and $1_a = 0$ otherwise. In this model, β_j will be the neuron's average activity in response to stimulus θ_j. The adaptive model fits neural responses across trials by the following linear model,

$$r_i \sim \sum_{j=1}^{8} \left(1_{x_i=\theta_j} \beta_j + \sum_{k=2}^{8} 1_{x_i=\theta_j, x_{i-1}=\theta_k} \beta_{jk} \right). \tag{116}$$

In this model, the neuron's mean activity in response to a stimulus with drift direction $x_i = \theta_j$ preceded by a stimulus with drift direction $x_{i-1} = \theta_1$ is β_j. The β_{jk}'s then model how this activity changes relative to β_j if the current trial's stimulus is instead preceded by a stimulus with drift direction $x_{i-1} = \theta_k$. For sessions with stimuli of multiple contrasts, the contrast in the table refers to that of the preceding stimulus (i.e., that in trial $i - 1$ of the *adapting* stimulus). As these models are nested (i.e., setting $\beta_{jk} = 0$ for all j and k turns the adaptive model into a non-adaptive one), we used an F-test to test the null hypothesis that the non-adaptive model fits the data better than the adaptive model. The above table shows the percentage of neurons for which this null hypothesis could be rejected at the $p = 0.05$ significance level. We performed a one-sided binomial test (H_0: fraction = 5%, testing for fraction $> 5\%$) to determine if any of the observed fractions are unlike to have arisen by chance, and found that none are significantly above 5% (one-sided $p > 0.124$ for all sessions/mice) [16]. This made us conclude that none of our datasets featured significant adaptation effects.

Mouse	Contrast	Avg.	Session A	B	C	D	E	F	G
1	10%	0.12 ± 0.02	0.14	0.11					
2	10%	0.07 ± 0.03	0.11	0.04					
3	10%	0.16 ± 0.02	0.22	0.20	0.09	0.13	0.14		
4	10%	0.12 ± 0.01	0.10	0.11	0.12	0.10	0.13	0.13	0.16
5	10%	0.22 ± 0.06	0.12	0.16	0.20	0.40			
	25%	0.23 ± 0.03	0.19	0.20	0.22	0.32			
6	10%	0.20 ± 0.02	0.16	0.23	0.22				
	25%	0.22 ± 0.01	0.20	0.23	0.24				

Supplementary Table 2: Average Fisher information per neuron in $rad^{-2}/neuron$, across all sessions/mice, averaged across all $\delta\theta = 45^\circ$ discriminations. The average Fisher information was computed from the Fisher information scaling for trial-shuffled data that removed across-neuron correlations. For individual neurons, it can be computed by $2\left(\langle r|\theta_1\rangle - \langle r|\theta_2\rangle\right)^2 / \left(\delta\theta^2\left(\text{var}\left(r|\theta_1\right) + \text{var}\left(r|\theta_2\right)\right)\right)$, where $r|\theta_j$ is the neural response to stimulus θ_j. The *Avg.* column provides the average across sessions (mean $\pm$ 1 SEM).

6 Supplementary Figures

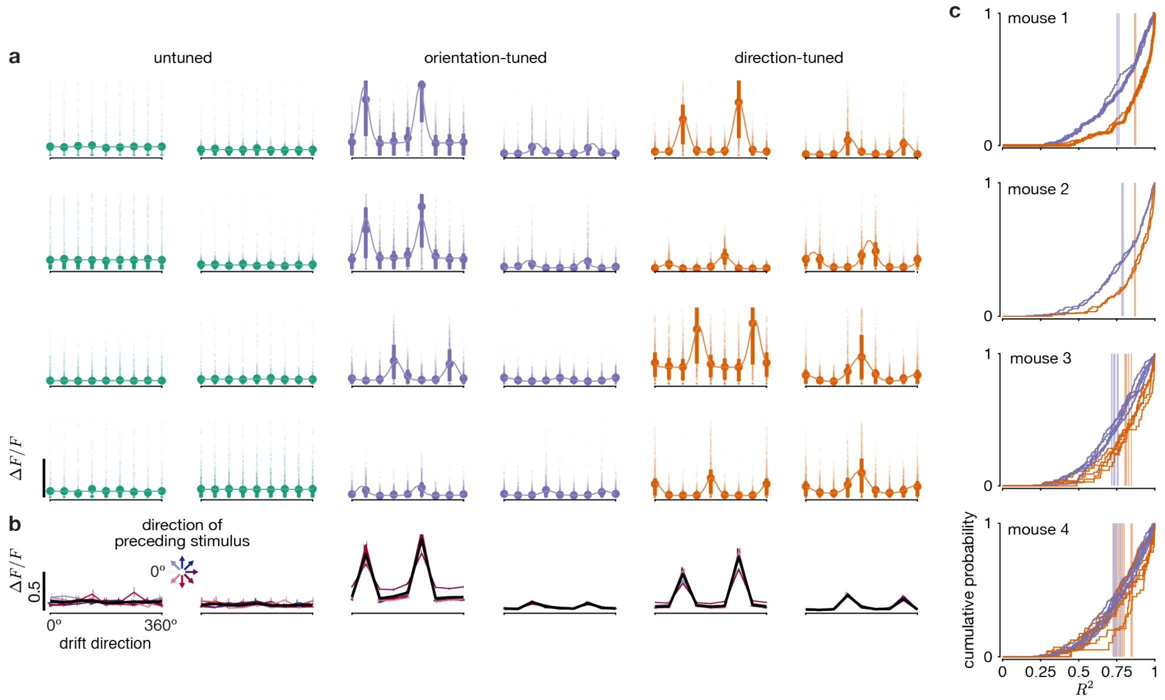

Supplementary Figure 1: Example tuning curves, absence of adaptation, and fitted tuning curve R^2's, for 10% contrast trials. (a) Eight examples of untuned, orientation-tuned, and direction-tuned neurons. We defined direction-tuned neurons (see Method) as having significantly higher responses for the tuned direction than the opposite direction. Orientation-tuned neurons are those for which this difference is not significant. The response of untuned neurons is not significantly modulated by drift direction. The pale, small dots show responses in individual trials. The large dots show mean responses for each drift direction, and the solid, vertical lines connect the 25th and 75th percentile. The pale lines show the fitted tuning curves. See Methods for how tuning was determined. Plots are truncated at $\Delta F/F = 1$. (b) Example direction tuning averaged across all trials (black), and across trials following a stimulus of a specific drift direction (colors, mean $\pm$ 1SEM, error bars horizontally shifted for visibility), demonstrating little to no adaptation to the preceding stimulus (see also Supplementary Supplementary Table 1). The shown example neurons are the same as in the first row of (a). (c) The cumulative distribution of coefficients of variations R^2 for different mice (rows) and sessions (line) for orientation-tuned (purple) and direction-tuned (orange) neurons. The pale vertical lines show the average R^2 for each session and neuron type. The R^2 for untuned neurons is not shown, as it is, by definition, $R^2 = 0$. (a) and (b) used data from one session of mouse 1 whose corresponding R^2 values are shown in bold in (c). All data at 0° is replicated at 360° to show the tuning curve across all possible drift directions. Note that fitted tuning curves were not used to estimate information, and are provided for reference only.

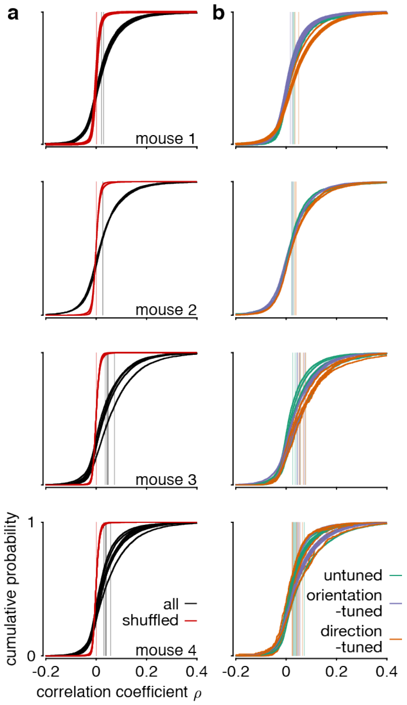

Supplementary Figure 2: Pairwise noise correlations across all neurons, and for neurons with specific tuning. (a) The cumulative distribution of pairwise noise correlations across all neurons collected within individual sessions (black lines) for mice 1-4 (rows). For reference, we also show these noise correlations for trial-shuffled data (red) that, on average, removes noise correlations. (b) The cumulative distribution of pairwise noise correlations for pairs of neurons with specific tuning (colors). See Methods for how tuning was determined. In both (a) and (b), the vertical lines show the mean pairwise correlations for the respective session and type of analysis. These mean correlations where comparable to those found in previous studies [17]. For mouse 1, the bold lines correspond to the session for which tuning curve examples are shown in Supplementary Figure 1(a)/(b). Note that the average pair-wise correlations were not used to estimate information, and are provided for reference only.

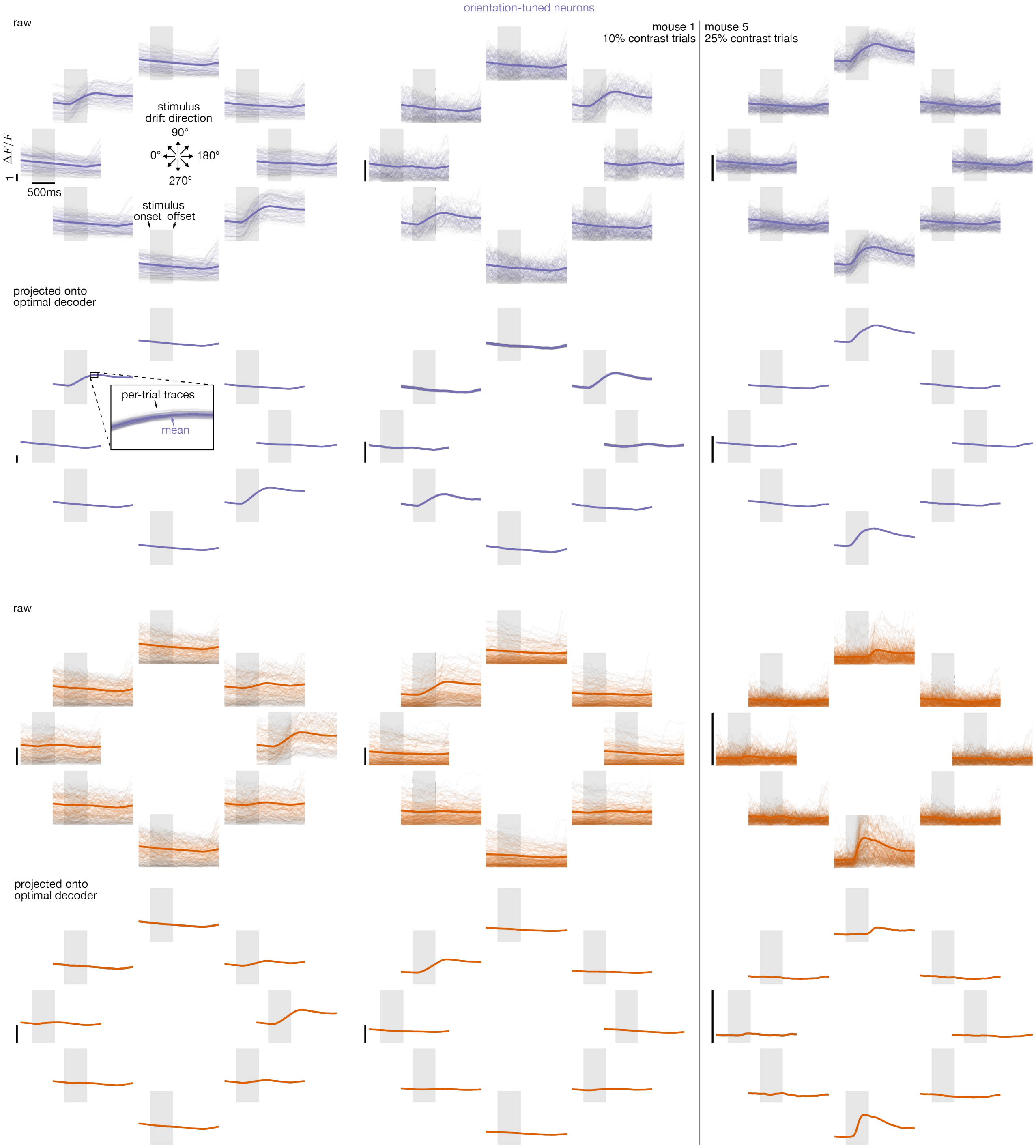

Supplementary Figure 3: Examples of raw $\Delta F/F$ traces and traces projected onto the optimal decoder. Each circle of 8 panels shows 200 raw $\Delta F/F$ time-course examples (thin lines) as well as their mean (thick line) for one example neuron, grouped by different stimulus drift directions (different panels; grey-shaded area = stimulus presentation period; black horizontal bar = 1 $\Delta F/F$). The top six panel circles show three orientation-tuned example neurons, and the bottom six panel circles represent three direction-tuned example neurons (left two columns: mouse 1, all trials 10% contrast; right column: mouse 5, only 25% contrast trials; see Supplementary Figure 1 for definition of orientation/direction-tuning). The top row in each neuron group shows the raw traces, and the bottom row shows the same traces projected onto the optimal decoder, $\mathbf{w} \propto \mathbf{\Sigma}^{-1}\mathbf{f}'$. The low variability of per-trial trace examples after this projection (frequently obscured by the across-trial mean; see enlarged inset) illustrates that most variability of the raw traces does not impact information, as it is orthogonal to the signal direction. The optimal decoder was for each stimulus drift direction computed (from temporally deconvolved traces) as the best discriminator between this and the next-closest drift direction.

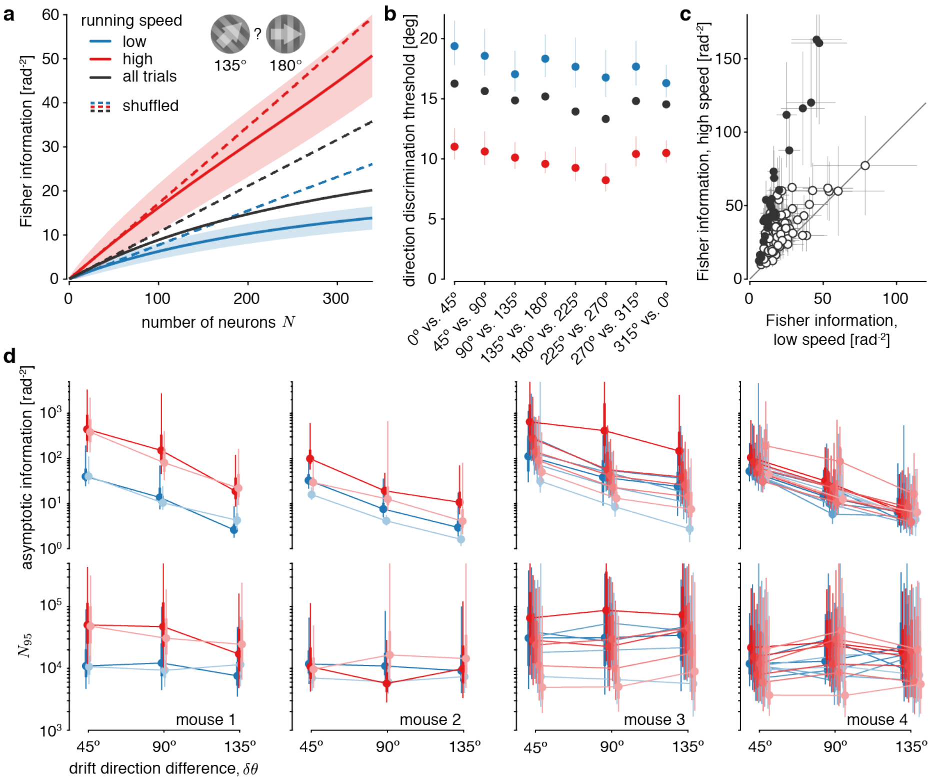

Supplementary Figure 4: Impact of running speed on information. For each session, we grouped trials into low (blue) and high (red) running speed by (i) subsampling trials to ensure a similar running speed distribution across all drift directions, and (ii) performing a median split by running speed for each drift direction. To maximize the number of analyzed trials, we did not aim to achieve the same average running speeds within each speed group across sessions — these average running speeds within each group might thus differ across sessions. Furthermore, mice 3 and 4 were overall running less than mice 1 and 2. (a) Information increases more rapidly with population size for higher running speeds (same mouse/session as in Supplementary Figure 3c; mean $\pm$ 1SD across random orderings of neurons within the population). The black line shows the mean information growth across all trials (as in Supplementary Figure 3c). The dashed lines show trial-shuffled data that removes pairwise noise correlations, and illustrate that, in all cases, these correlations lower information across all population sizes. (b) The drift direction discrimination threshold (80% correct) inferred from the information estimated in the recorded population is consistent across different drift direction pairs with $\delta\theta = 45°$, and is lower for high running speeds. The black dots show the inferred thresholds across all trials (as in Supplementary Figure 3f). (c) Higher running speed increases information in the recorded population. Each dot (mean $\pm$ 1SD of information estimate; filled = significant increase, bootstrap, p<0.05) shows the information estimated for one discrimination with $\delta\theta = 45°$. Across all sessions, higher running speed significantly increased information ($t_{63} = 6.69$, two-sided $p \approx 7 \times 10^{-9}$, across non-overlapping $\delta\theta = 45°$ discriminations). (d) Both estimated asymptotic information and N_{95} appear impacted by running speed (lines connect median estimates of individual sessions; horizontally shifted to ease comparison; posterior densities as is Supplementary Figure 4c). Across sessions, we found a significant increase in asymptotic information (signed-rank on median estimates; $\delta\theta = 45°$: $z = 2.64$, two-sided $p \approx 8.36 \times 10^{-3}$; $\delta\theta = 90°$: $z = 2.69$, two-sided $p \approx 7.17 \times 10^{-3}$; $\delta\theta = 135°$: $z = 3.36$, two-sided $p \approx 7.76 \times 10^{-4}$; not adjusted for multiple comparisons across $\delta\theta$), but not N_{95} (signed-rank on median estimates; $\delta\theta = 45°$: $z = 0.672$, two-sided $p \approx 0.501$; $\delta\theta = 90°$: $z = 1.14$, two-sided $p \approx 0.255$; $\delta\theta = 135°$: $z = 1.500$, two-sided $p \approx 0.134$; not adjusted for multiple comparisons across $\delta\theta$) with running speed.

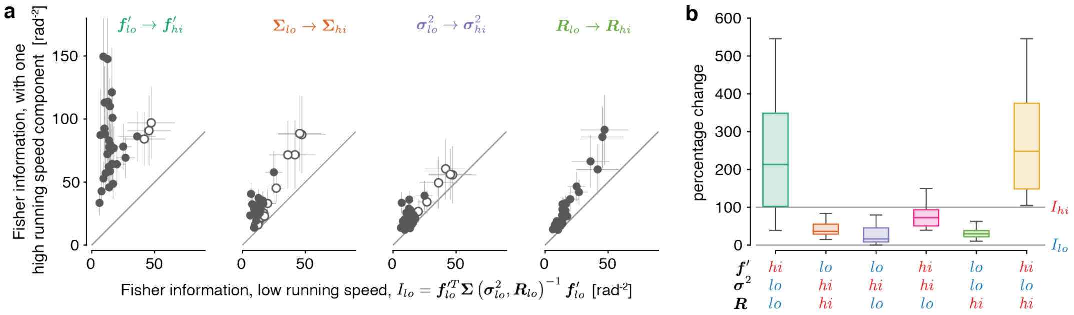

Supplementary Figure 5: Information boost due to increased running speed results from a combination of multiple factors. To identify which changes to the population response statistics are responsible for the boost in information for increased running speeds (see Supplementary Figure 4), we focused on $\delta\theta = 45°$ discriminations in each session for which we observed a significant information increase (filled dots in Supplementary Figure 4c). We focused on three factors: (i) a change in mean responses, $\mathbf{f}'$, (ii) a change in per-neuron noise variances, σ^2, and (iii) a change in pairwise noise correlations, $\mathbf{R}$. Per-neuron variances and pairwise noise correlations completely determine the noise covariance matrix $\mathbf{\Sigma}\left(\sigma^2, \mathbf{R}\right)$. For each considered discrimination, we computed these factors separately for trials in which the running speed was low, denoted $\cdot_{lo}$, and in which it was high, denoted $\cdot_{hi}$. With this notation, the information in the recorded population for low and high running speeds, as shown in Supplementary Figure 4, is given by $I_{lo} = \mathbf{f}_{lo}'^T \mathbf{\Sigma}_{lo}^{-1} \mathbf{f}_{lo}'$ and $I_{hi} = \mathbf{f}_{hi}'^T \mathbf{\Sigma}_{hi}^{-1} \mathbf{f}_{hi}'$, where we have used the short-hand notation $\mathbf{\Sigma}_x = \mathbf{\Sigma}\left(\sigma_x^2, \mathbf{R}_x\right)$ with $x \in \{lo, hi\}$. (a) All factors boost information individually (mean $\pm$ 1SD of information estimate; filled = significant increase, bootstrap, p<0.05). To see the impact on information if only a single of these factors changes, we compared I_{lo} for all considered discriminations to the information when a single factors (except for $\mathbf{\Sigma}$, for which we changed both σ^2 and $\mathbf{R}$) was changed from $\cdot_{lo}$ to $\cdot_{hi}$. Across all considered discriminations we observed a significant information increase due to all factors (two-sided t-test, $t_{32} < -7.22$, $p < 3.34 \times 10^{-8}$). (b) Relative information boost on the I_{lo} — I_{hi} scale. To see how the information due to separate factors compares to that due to all factors, we computed the percentage of information boost for each considered discrimination and factor combination (different bars) where the information boost lies on the scale from I_{lo} (0%) to I_{hi} (100%). Each box plot shows the median, the 25% and 75% percentiles (box) and the extremes (whiskers) of the percentage information boost for a given combination of factors across considered discriminations. The relative information boost can exceed 100% in cases in which changing the remaining $\cdot_{lo}$ factor to $\cdot_{hi}$ results in a drop of information, as seen for $(\mathbf{f}'_{hi}, \mathbf{\Sigma}_{lo})$ and $\left(\mathbf{f}_{hi}', \sigma_{lo}^2, \mathbf{R}_{hi}^2\right)$. Both in combination show that the change of $\mathbf{f}'$ from $\mathbf{f}_{lo}'$ to $\mathbf{f}_{hi}'$, which results in an information boost beyond I_{hi}, is compensated by a change of the per-neuron variances σ^2 from σ_{lo}^2 to σ_{hi}^2. n=4 mice with a total of 16 sessions, resulting in 128 discriminations that were utilized for this analysis.

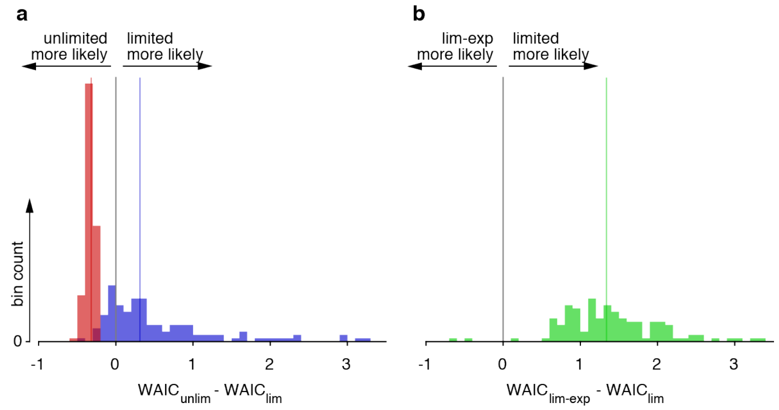

Supplementary Figure 6: Model comparison of different information scaling models. Both panels show histograms of differences in the Watanabe-Akaike Information Criterion (WAIC) for two different models fitted to the measured information scaling curves across all eight discriminations with $\delta\theta = 45^\circ$, sessions, and mice. (a) shows the WAIC difference for fitting a model that assumes no information limitation (*unlim*) to one that does (*lim*), for regular (blue) and shuffled (red) data. For regular data this difference is in most cases positive, indicating that the information-limiting model fits the data better. In fact, even for individual negative WAIC differences, the average across all eight WAIC difference within a session remains positive. For shuffled data, a model assuming no information limitation fits the data better in all instances. This confirms that our model comparison is not biased towards the model assuming limited information. (b) shows the WAIC difference for fitting two models that assume limited information (see Sec. 2.2), one with linear scaling of the non-limiting component (*lim*), and one assuming initial supralinear scaling of that component (*lim-exp*). The latter only fits the data better in few instances. In those, the average WAIC difference across all discriminations within that session is nonetheless positive. The colored lines in (a) and (b) show the median WAIC difference across all comparisons.

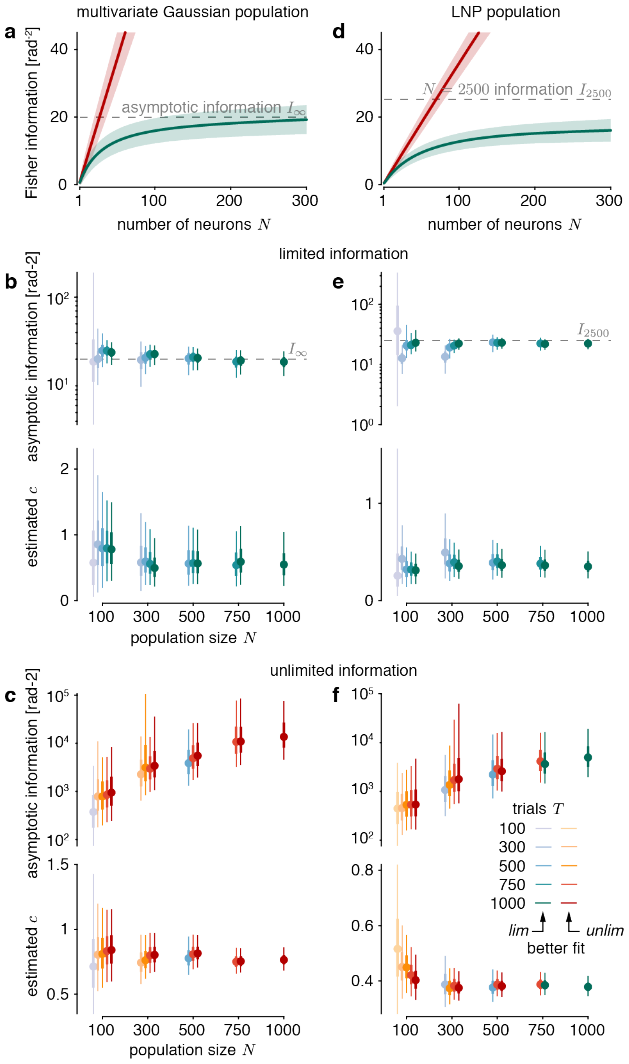

Supplementary Figure 7: Recovering asymptotic information from simulated population activity. We simulated neural population activity, using either a multivariate Gaussian population model (a-c; see Sec. 4.1 for details) or a linear-nonlinear Poisson model (d-f; see Sec. 4.2 for details) and fitted a linear scaling (*unlim*) and a limited information scaling model (*lim*). For each model type, we generated two large datasets (limited information and unlimited information; $\delta\theta = \theta_2 - \theta_1 = 45^\circ$ in both cases) and then subsampled neurons and trials to perform the fits. (a,d) Example information scaling for $N = 300$ and $T = 500$ (mean ± 1SD information estimation; green/red = limited/unlimited information). For the Gaussian model we could specify the asymptotic information I_∞ (dashed grey line). For the LNP model we estimated it from the information I_{2500} at $N = 2500$ neurons. (b-c,e-f) Estimated asymptotic information and non-limiting information scaling for the *lim* model from data with different population sizes N and numbers of trials T per stimulus. The posterior estimates are shown as in Supplementary Figure 4c in the main text. Blue/green and orange/red colors indicate a better fit by the *lim* and *unlim* model (WAIC for model comparison), respectively. Asymptotic information is well-estimated by the *lim* model (b,e), and more certain for larger N and T. Model comparison in most cases (28 out of 30 for Gaussian model, 26 out of 30 for LNP model) correctly identifies if information was limited or unlimited (colors).

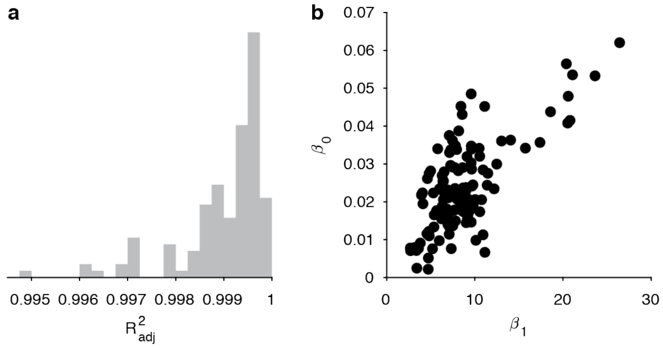

Supplementary Figure 8: Statistics of a linear fit $I_N^{-1} = \beta_0 + \beta_1 N^{-1}$ across all eight discriminations with $\delta\theta = 45°$, sessions, and mice. (a) The adjusted R^2 is close to one for all fits. (b) Both intercept, β_0, and slope, β_1, are significantly above zero for all discriminations. The plot shows these intercepts with 95% CIs, which are obscured by the dots.

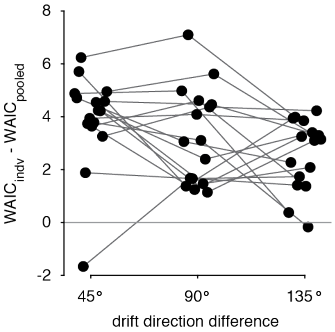

Supplementary Figure 9: Model comparison of per-discrimination fits vs. pooled fits across multiple discriminations. The figure shows for each session (individual sessions connected by grey lines; horizontally jittered for clarity) the WAIC difference of fitting the information scaling of individual discriminations (*indv*) vs. fitting all of these discriminations simultaneously (*pooled*). The mostly positive WAIC differences, preferring pooled fits, confirm that the information scaling across different discriminations with the same drift direction difference $\delta\theta$ were exceedingly similar. The tested discriminations were $45°$ vs. $90°$, $135°$ vs. $180°$, $225°$ vs. $270°$, and $315°$ vs. $0°$ ($\delta\theta = 45°$); $45°$ vs. $135°$, $90°$ vs. $180°$, $225°$ vs. $315°$, and $270°$ vs. $0°$ ($\delta\theta = 90°$); and $45°$ vs. $180°$, $90°$ vs. $315°$, and $225°$ vs. $0°$ ($\delta\theta = 135°$). The WAIC differences for $\delta\theta = 315°$ had overall smaller magnitudes, as they pooled across three rather than four discriminations.

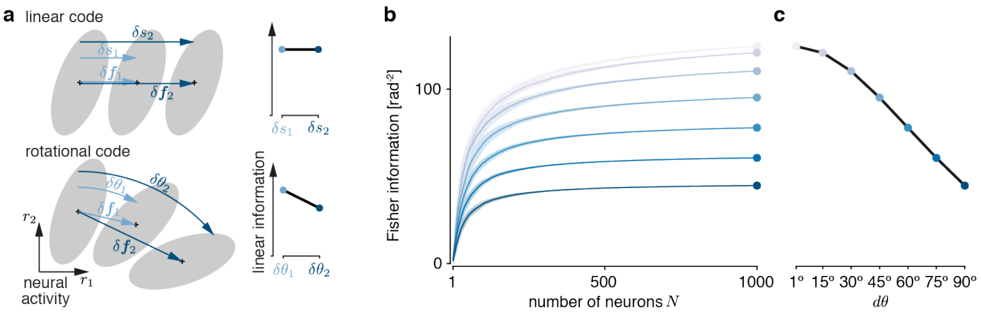

Supplementary Figure 10: Linear Fisher information is expected to drop with increasing $\delta\theta$. (a) Generalized linear Fisher information measures how easy it is to discriminate two stimuli from the population responses they evoke. This discriminabiliy is measured by the performance of a linear discriminator, normalized by the stimulus difference (δs or $\delta\theta$). For population responses (dots = mean population activity for one stimulus, shaded areas = 1SD of the noise covariance; $\delta \mathrm{f}_i$ = difference in mean population activity for different δs_i / $\delta\theta_i$) whose mean response changes linearly with the stimulus s, this information remains unchanged when δs changes (top; δs_1 vs. δs_2). Population activity that encodes a circular stimulus θ is bound to violate this linearity, and its associated linearly decodable information drops with an increase in $\delta\theta$ (bottom; $\delta\theta_1$ vs. $\delta\theta_2$). This occurs also if a non-linear decoder that accounts for the circularity of θ would recover the same information, irrespective of $\delta\theta$, and is not a bug of the linear decoder, which nonetheless correctly identifies all linearly decodable information (that drops with $\delta\theta$). (b) We demonstrate this effect by simulating V1 population in response to oriented Gabor pattern, and estimate the information encoded about their orientation. We show how information grows with population sizes for stimulus pairs with different $\delta\theta$ (colors; mean $\pm$ 1SD across different orders with which neurons are added to the population). (c) The information at $N = 1000$, which we use as a proxy for I_∞, drops with $\delta\theta$, for the reason illustrated for the rotational code in (a). Details of the simulations to generate (b) and (c) are described in Sec. 4.2. The simulations quantify information about oriented Gabor pattern rather than the drift direction of drifting gratings, and so should only be qualitatively compared to the data in the main text.

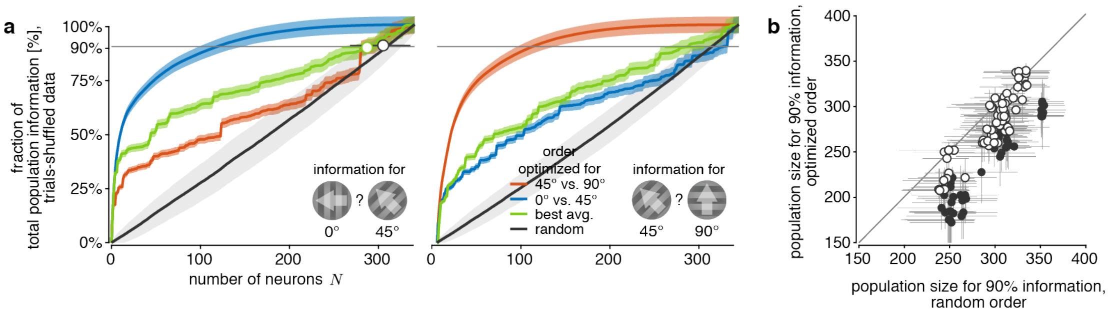

Supplementary Figure 11: Same as Supplementary Figure 5, but for trial-shuffled data. As in Supplementary Figure 5, we asked if a subpopulation appears to encode a disproportionate amount of information across all stimulus drift directions. In contrast to Supplementary Figure 5, we here removed the impact of noise correlations by, for each neuron and drift direction, randomly shuffling the trial identity. (a) Both panels show that information increase in the recorded population depends on the order with which neurons are added to the population (colors). The panels differ in the considered drift direction discrimination (left: $0°$ vs. $45°$; right: $45°$ vs $90°$). The neuron order was optimized by incrementally adding the neuron that resulted in the largest information increase for a $0°$ vs. $45°$ (blue) or $45°$ vs $90°$ (orange) drift direction discrimination, or largest average increase across all discriminations with $\delta\theta = 45°$ (green). The optimal ordering for the $0°$ vs. $45°$ was also applied to the $45°$ vs $90°$ discrimination (blue line in right panel) and vice versa (orange line in left panel). The average information increase across random orders (black) is shown as baseline reference. Shaded error regions illustrate the uncertainty (mean $\pm$ 1SD) due to limited numbers of trials (all curves), and variability across random orderings (black only). The black and green open circle (bootstrapped median $\pm$ 95% CI) show the population sizes required to capture 90% of the information in the recorded population for the associated orderings. (b) Plotting population sizes required to capture 90% of the information in the recorded population (bootstrapped median $\pm$ 95% CI) for random ordering vs. orderings optimized to maximize average information across all discriminations revealed a significant difference between the two orderings for some datasets (filled dots). Each dot reflects one discrimination for one session. The difference in population sizes was also significant across all datasets (t-test, $t_{63} = 9.541$, two-sided $p = 6.1 \times 10^{-14}$, across non-overlapping $\delta\theta = 45°$ discriminations).

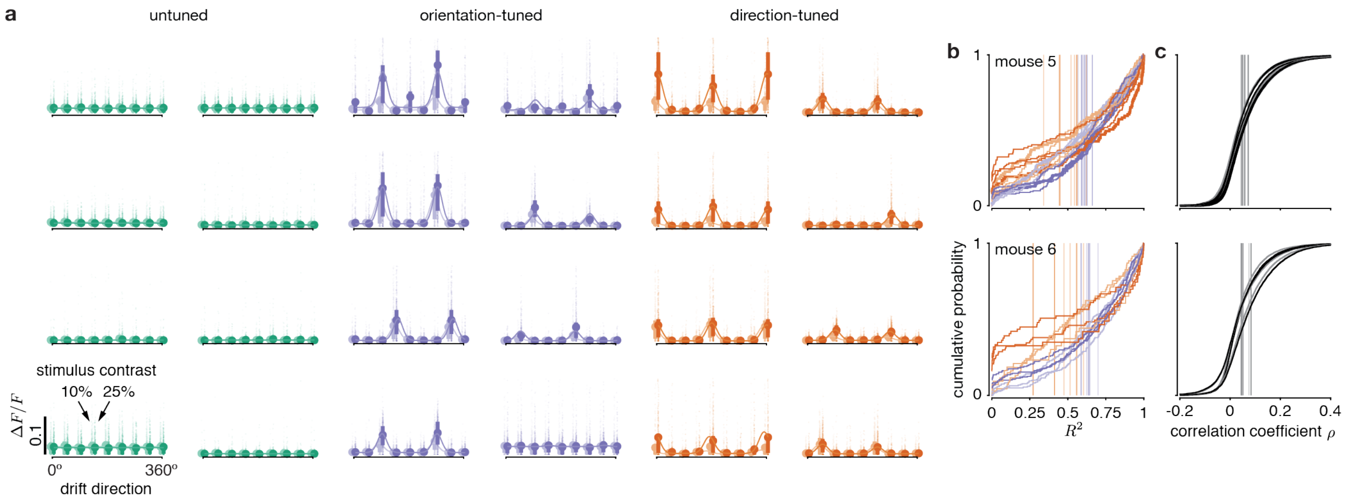

Supplementary Figure 12: Example tuning curves, tuning curve R^2's, and pair-wise correlations, for the 10% and 25% contrast trials of mice 5 and 6. We determined the tuning type (untuned, orientation-tuned, or direction-tuned) of each neuron by fitting the 25% contrast trials only, and chose the best-fit tuning curve function $f_{25}(\theta)$ (functional form depends on tuning type) as described in Methods. We then jointly fit the 10% and 25% contrast data by using the previously determined $f_{25}(\theta)$ function to fit the 25% contrast trials, and $f_{10}(\theta) = a + bf_{25}(\theta)$ to fit the 10% contrast trials. To do so, we jointly adjusted the parameters of the f_{25} function, as well as a and b. Except for untuned neurons, this resulted in tuning curve fits with fewer parameters than if we would have fitted the tuning curves for each contrast level separately. Bayesian model comparison that accounted for the different numbers of parameters revealed that, for most neurons, this joint fit across both contrast levels explained the data better than separate fits for each contrast level ($\text{BIC}_{\text{joint}} < \text{BIC}_{\text{separate}}$; mouse 5: direction-tuned 76.54% (310 of 405 neurons), orientation-tuned 94.21% (683 of 725 neurons); mouse 6: direction-tuned 70.35% (140 of 199 neurons), orientation-tuned 92.41% (597 of 646 neurons)). (a) Eight examples of untuned, orientation-tuned, and direction-tuned neurons. The pale, small dots show responses in individual trials. The large dots show mean responses for each drift direction, and the solid, vertical lines connect the 25th and 75th percentile. The pale lines shows the fitted tuning curves. Each panel shows data for both 10% and 25% contrast trials. Data, raw, and fitted tuning curves are darker for 25% contrast trials and slightly shifted to the right. Plots are truncated at $\Delta F/F = 0.2$. (b) Cumulative distributions of coefficients of variation R^2 for different mice (rows) and sessions (line) for orientation-tuned (purple) and direction-tuned (orange) neurons for 25% (dark) and 10% (bright) contrast trials. The R^2 values are computed separately for each contrast level, even though the tuning curves were fit jointly across the two contrast levels. (c) The cumulative distribution of pairwise noise correlations for pairs of neurons for 25% contrast (dark) and 10% contrast (bright trials), shown separately for each session. (a) used data from one session of mouse 5 whose corresponding R^2 values are shown in bold in (b) and (c). All data at 0° is replicated at 360° to show the fitted tuning curve across all possible drift directions. The pale vertical lines in (b) and (c) show the average R^2 and correlation coefficients for each sessions, tuning type, and contrast level. The observed mean correlations were comparable to those found in previous studies [17]. Note that fitted tuning curves and average pair-wise correlations were not used to estimate information, and are provided for reference only.

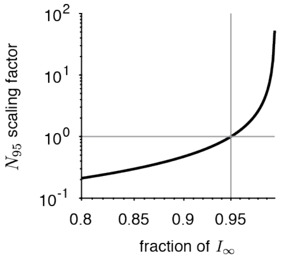

Supplementary Figure 13: The scaling of the estimated population size with the fraction of asymptotic information. Let N_a denote the population size required to encode $a\%$ of the total asymptotic information, I_∞. Changing a results in a simple re-scaling of N_a. This figures illustrates this re-scaling for different a, using N_{95} as a base measure. For example, if we would be interested in N_{90} instead of N_{95}, we would read off the scaling factor for 90%, and would re-scale the reported N_{95} to get estimates for N_{90}.

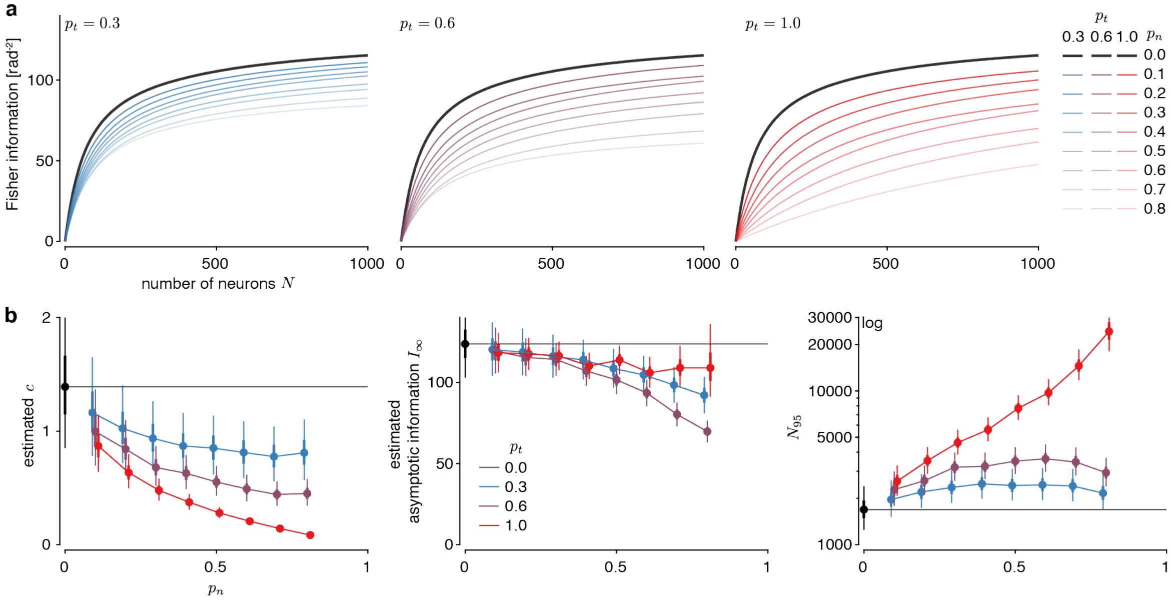

Supplementary Figure 14: Effects of modeled eye movement on information scaling. We assessed the effects of eye movements on information scaling using a simple eye movement model (see Sec. 2.4 for details). In this model, in a fraction of trials p_t the activity of a fraction of neurons p_n was set to zero. (a) We simulated population activity by adding a Poisson step to the model described in Sec. 4.2 to simulate spike counts in a 500ms window in a population of $N = 1000$ neurons in response to oriented Gabor pattern, and estimated bias-corrected linear Fisher information for different population sizes, different p_t's (panels/colors), and different p_n's (color shading). As can be seen, information grows more slowly with number of neurons N with increasing p_t and increasing p_n. (b) We fitted our information scaling model to the information scaling curves in (a) to estimate non-asymptotic scaling c (left) and asymptotic information I_∞ (center). We used these estimates to, in turn, estimate the number of neurons N_{95} required to encode 95% of asymptotic information (right). The posterior estimates are shown as in Supplementary Figure 4c in the main text. As predicted by our theory (see Sec. 2.4), asymptotic information scales with $p_t(1-p_t)$: it hardly drops if $p_t = 1$, and drops most strongly for $p_t = 0.6$. This also confirms that $\boldsymbol{f}_{ori}$ is most likely not perfectly aligned to $\boldsymbol{f}'_{ori}$ in our simulations. The estimated c, in contrast, drops monotonically with both an increase in p_t and in p_n. In combination, this yields an overestimation of N_{95} that grows with both p_t and p_n.

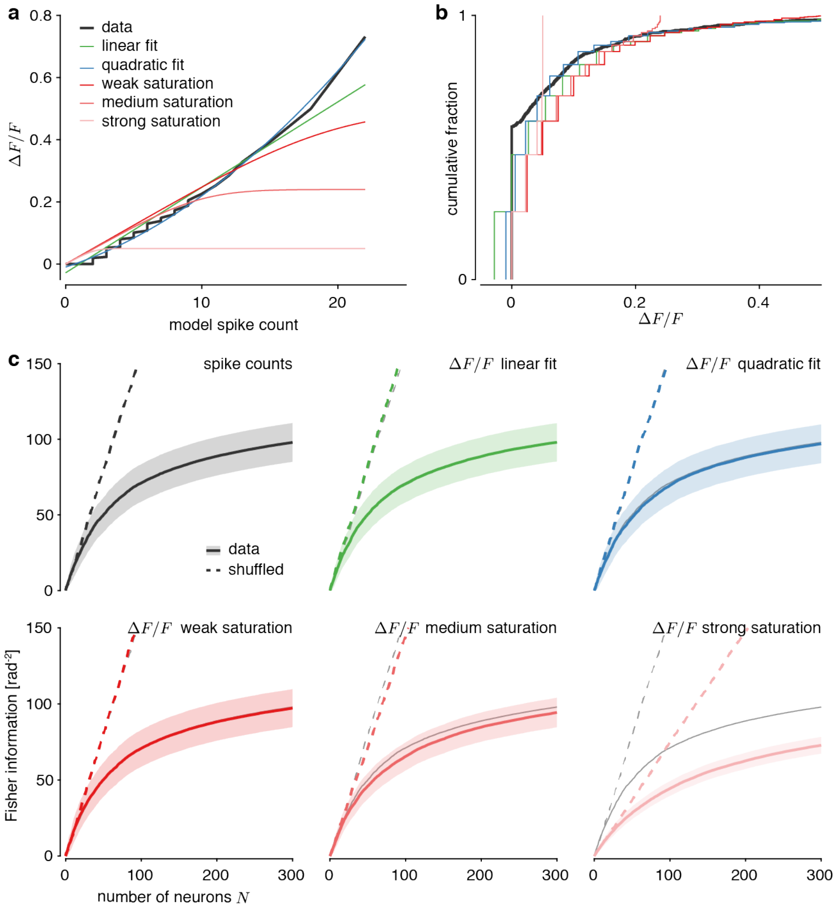

Supplementary Figure 15: A non-linear mapping between spike counts and $\Delta F/F$ signal does not qualitatively impact our results. We added a Poisson step to the model described in Sec. 4.2 to simulate spike counts in a 500ms window in a population of $N = 300$ neurons in response to oriented Gabor pattern. We then used linear and non-linear functions that map these spike counts to $\Delta F/F$ signals that were in turn used to estimate how information scales with population size. (a) The different utilized functions for mapping per-neuron spike counts to $\Delta F/F$ signals. The black line connects the percentiles (from the 1st to the 99th percentile) of the distribution of simulated spike counts to those of the distribution of $\Delta F/F$ responses of the data of session 1 of mouse 1. The near-linear relationship indicates that a linear remapping of these spike counts will well-replicate the observed $\Delta F/F$ distribution. We fitted this relationship with both a linear (green) and a quadratic function (blue). We furthermore tested various saturating functions (different shades of red, weak: $0.5\tanh\left((0.5r)^2\right)^{1/2}$, medium: $0.24\tanh\left((0.1r)^2\right)^{1/2}$, strong: $0.05\tanh\left((0.45)^2\right)^{1/2}$, where r is the spike count) to simulate the scenario in which the $\Delta F/F$ response saturates for higher spike counts. (b) The cumulative distribution function of the $\Delta F/F$ distributions of the data of session 1 of mouse 1 (black), the linear (green) and quadratic (blue) model, and the different saturating models (shades of red). In particular the strongly saturating model results in significant deviations from the data. (c) Information scaling computed from the $\Delta F/F$ signal (except for the black & grey lines, which are based on spike counts) for the different mappings between spike counts and $\Delta F/F$. The dashed lines show results when trial-shuffling the spike count data before mapping it to $\Delta F/F$ signals to destroys the spike count noise correlations (see Supplementary Figure 3). Any eventual difference between Fisher information computed from spike counts (black) and the linear model (green) are due to numerical precision, as invertible linear transformations, as used here, do not change the Fisher information. Most importantly, trial-shuffled spike count data yields linear information scaling even after non-linear mappings, as these mappings do not introduce new noise correlations. Similarly, the information scaling of non-shuffled data saturates even after perturbing the spike counts with a non-linear mapping, as this mapping does not remove the noise correlations. While strong non-linear mappings might lower our information estimates, they do not impact our finding that information saturates.

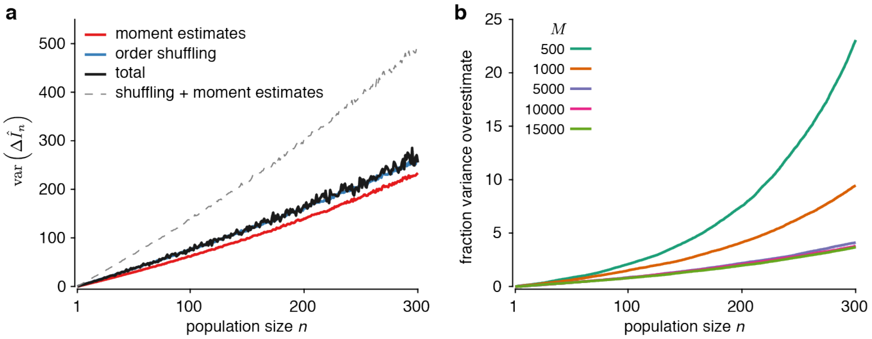

Supplementary Figure 16: The variance and covariance of Fisher information scaling. We simulated virtual populations of different sizes M as described in Sec. 4.1, yielding $\mathbf{f}'$ and $\mathbf{\Sigma}$ for each M. (a) To demonstrate that the variance in the Fisher information increase estimate due to shuffling well-approximates the combined variance due to population subsampling and due to estimating the moments from a finite number of trials, we generated one population with $M = 10,000$ neurons. We in turn drew 100 empirical moments, $\gamma \sim \mathcal{N}\left(\mathbf{f}', 2\mathbf{\Sigma}/(T\delta\theta)^2\right)$ and $\mathbf{\Omega} \sim \mathcal{W}\left(\mathbf{\Sigma}/(2T-2), 2T-1\right)$, corresponding to estimating these moments from $T = 1,000$ trials each for two drift directions separated by $\delta\theta = 45°$. We additionally subsampled $N = 300$ neurons of the full population ten times, resulting in ten $i_{1:N}$ neuron indices, and, for each $i_{1:N}$, containing a fixed set of neurons, shuffled their order 100 times, resulting in 100 $j_{1:N}$ per $i_{1:N}$. For the empirical moments, we computed the Fisher information increase for each subsampled, shuffled population $j_{1:N}$, resulting in 10^6 estimates for each population size $n \in 1, \ldots N$. The figure shows the variance due to shuffling only (blue, averaged over different subsamples and empirical moments), and due to empirical moments only (red, averaged over different subsamples and shuffles). As comparison, we computed the total variance of the same estimate across 100 subsampled populations with $N = 300$ neurons for each set of empirical moment (black; variance across 10^4 estimates), which is the variance we aim to estimate. As the plot shows, the variance due to shuffling well-approximates this total variance. A naïve sum of the variance due to empirical moments and shuffling (grey dashed) would over-estimate the total variance. (b) To estimate the degree by which the variance of the Fisher information increase, var $\left(\Delta \hat{I}_n\right)$, is overestimated when ignoring the negative correlations across different $\Delta \hat{I}_n$'s, we generate populations of different sizes, M, and their associated moments. For each population, we then estimated the covariance cov $\left(\Delta \hat{I}_n, \Delta \hat{I}_m\right)$ across 1,000 different subsamples $i_{1:N}$ of populations of $N = 300$ neurons. In turn, we estimated the Fisher information variance once when taking into account this covariance, var $\left(\hat{I}_n\right) = \sum_{j=1}^{n} \left(\text{var}\left(\Delta \hat{I}_j\right) + 2\sum_{k=1}^{j-1} \text{cov}\left(\Delta \hat{I}_k, \Delta \hat{I}_j\right)\right)$, and once when not doing so, $\tilde{\text{var}}\left(\hat{I}_n\right) = \sum_{j=1}^{n} \text{var}\left(\Delta \hat{I}_j\right)$. The plot shows the resulting fraction $\left(\tilde{\text{var}}\left(\Delta \hat{I}_n\right) - \text{var}\left(\Delta \hat{I}_n\right)\right) / \text{var}\left(\Delta \hat{I}_n\right)$ for different n and M as an average across ten different generated populations, and shows that the variance overestimate becomes smaller for larger populations.

Supplementary References

[1] Rubén Moreno-Bote, Jeffrey Beck, Ingmar Kanitscheider, Xaq Pitkow, Peter Latham, and Alexandre Pouget. Information-limiting correlations. *Nature Neurosience*, 17(10):1410–1417, 2014. doi: 10.1038/nn.3807.

[2] Deep Ganguli and Eero P. Simoncelli. Efficient sensory encoding and bayesian inference with heterogeneous neural populations. *Neural Computation*, 26:2103–2134, 2014. doi: 10.1162/NECO_a_00638.

[3] Thomas M. Cover and Joy A. Thomas. *Elements of Information Theory*. Wiley Series in Telecommunications and Signal Processing. Whiley-Interscience, 2nd edition, 2006.

[4] Wei Ji Ma, Jeffrey M Beck, Peter E Latham, and Alexandre Pouget. Bayesian inference with probabilistic population codes. *Nature Neuroscience*, 9:1432–1438, 2006. doi: 10.1038/nn1790.

[5] James O. Berger. *Statistical decision theory and Bayesian analysis*. Springer Series in Statistics. Springer, 2nd edition, 1993.

[6] David Marvin Green and John A. Swets. *Signal detection theory and psychophysics*. Peninsula Pub, 1989.

[7] Bruno B. Averbeck and Daeyeol Lee. Effects of noise correlations on informatino encoding and decoding. *Journal of Neurophysiology*, 95:3633–3644, 2006. doi: 10.1152/jn.00919.2005.

[8] Yuzhi Chen, Wilson S. Geisler, and Eyal Seidemann. Optimal decoding of correlated neural population responses in the primate visual cortex. *Nature Neuroscience*, 9(11):1412–1420, 2006. doi: 10.1038/nn1792.

[9] Ramon Nogueira, Nicole E. Peltier, Akiyuki Anzai, Gregory C. DeAngelis, Julio Martínez-Trujillo, and Rubén Moreno-Bote. The effects of population tuning and trial-by-trial variability on information encoding and behavior. *The Journal of Neuroscience*, 40(5):1066–1083, 2020. doi: 10.1523/JNEUROSCI.0859-19. 2019.

[10] James O. Berger. *Pattern recognition and machine learning*. Information Science and Statistics. Springer, 2011.

[11] Ingmar Kanitscheider, Ruben Coen-Cagli, Adam Kohn, and Alexandre Pouget. Measuring fisher information accurately in correlated neural populations. *PLoS Computational Biology*, 11(6):e1004218, 2015. doi: 10.1371/journal.pcbi.1004218.

[12] Richard A. Johnson and Dean W. Wichern. *Applied multivariate statistical analysis*. Pearson, 5th edition, 2007.

[13] Robb J. Muirhead. *Aspects of multivariate statistical theory*. Wiley, 2nd edition, 2005.

[14] Kazuhiro Suzuki, Dongvu Tonien, Kaoru Kurosawa, and Koji Toyota. Birthday paradox for multi-collisions. In Min Surp Rhee and Byoungcheon Lee, editors, *Information Security and Cryptology — ICISC 2006*, pages 29–40. Springer-Verlag Berlin Heidelberg, 2006. doi: 10.1007/11927587_5.

[15] Ingmar Kanitscheider, Ruben Coen-Cagli, and Alexandre Pouget. Origin of information-limiting noise correlations. *Proceedings of the National Academy of Sciences*, 112(50):E6973–E6982, 2015. doi: 10.1073/pnas. 1508738112.

[16] Ramon Nogueira, Juan M. Abolafia, Jan Drugowitsch, Emili Balaguer-Ballester, Maria Sanchez-Vives, and Rubén Moreno-Bote. Lateral orbitofrontal cortex anticipates choices and integrates prior with current information. *Nature Communications*, 8:14823, 2017. doi: 10.1038/ncomms1482.

[17] Marlene R Cohen and Adam Kohn. Measuring and interpreting neuronal correlations. *Nature Neurosience*, 14(7):811–819, 2011. doi: 10.1038/nn.2842.
