# Supplementary material for: Scaling of sensory information in large neural populations shows signatures of information-limiting correlations
Source: Nat Commun. 2021 Jan 20;12:473. doi: 10.1038/s41467-020-20722-y (PMC7817840; doi:10.1038/s41467-020-20722-y)
Supplement: Supplementary file 3 — Reporting Summary [file 41467_2020_20722_MOESM3_ESM.pdf]

nature research

Corresponding author(s): Jan Drugowitsch

Last updated by author(s): Nov 17, 2020

Reporting Summary

Nature Research wishes to improve the reproducibility of the work that we publish. This form provides structure for consistency and transparency in reporting. For further information on Nature Research policies, see Authors & Referees and the Editorial Policy Checklist .

Statistics

For all statistical analyses, confirm that the following items are present in the figure legend, table legend, main text, or Methods section.

n/a	Confirmed	
☐	☒	The exact sample size (*n*) for each experimental group/condition, given as a discrete number and unit of measurement
☐	☒	A statement on whether measurements were taken from distinct samples or whether the same sample was measured repeatedly
☐	☒	The statistical test(s) used AND whether they are one- or two-sided<br>*Only common tests should be described solely by name; describe more complex techniques in the Methods section.*
☒	☐	A description of all covariates tested
☐	☒	A description of any assumptions or corrections, such as tests of normality and adjustment for multiple comparisons
☐	☒	A full description of the statistical parameters including central tendency (e.g. means) or other basic estimates (e.g. regression coefficient) AND variation (e.g. standard deviation) or associated estimates of uncertainty (e.g. confidence intervals)
☐	☒	For null hypothesis testing, the test statistic (e.g. F, t, r) with confidence intervals, effect sizes, degrees of freedom and P value noted<br>*Give P values as exact values whenever suitable.*
☐	☒	For Bayesian analysis, information on the choice of priors and Markov chain Monte Carlo settings
☒	☐	For hierarchical and complex designs, identification of the appropriate level for tests and full reporting of outcomes
☒	☐	Estimates of effect sizes (e.g. Cohen's *d*, Pearson's *r*), indicating how they were calculated

Our web collection on statistics for biologists contains articles on many of the points above.

Software and code

Policy information about availability of computer code

Data collection	Visual stimuli were generated using PsychoPy v3.0.6 (Peirce, 2007) or Psychtoolbox-3 (for mice 1 and 2 only). The two-photon microscope hardware was controlled by ScanImage 2018 (Vidrio Technologies).
Data analysis	All analysis was performed using custom MATLAB (version R2018b) code. The MATLAB codes are available on GitHub from (?????), (https://github.com/HarveyLab/Acquisition2P_class), and (https://github.com/Selmaan/NMF-Source-Extraction)

For manuscripts utilizing custom algorithms or software that are central to the research but not yet described in published literature, software must be made available to editors/reviewers. We strongly encourage code deposition in a community repository (e.g. GitHub). See the Nature Research guidelines for submitting code & software for further information.

Data

Policy information about availability of data

All manuscripts must include a data availability statement. This statement should provide the following information, where applicable:

- Accession codes, unique identifiers, or web links for publicly available datasets
- A list of figures that have associated raw data
- A description of any restrictions on data availability

The datasets generated and analyzed during this study are available on Figshare from (???????)

Field-specific reporting

Please select the one below that is the best fit for your research. If you are not sure, read the appropriate sections before making your selection.

☒ Life sciences ☐ Behavioural & social sciences ☐ Ecological, evolutionary & environmental sciences

Life sciences study design

All studies must disclose on these points even when the disclosure is negative.

Sample size	Sample sizes were chosen to be in line with previous comparable work (e.g., Rumyantsev et al., 2020)
Data exclusions	No data were excluded.
Replication	No attempts at replication outside of this study were performed.
Randomization	Mice were randomly assigned to experimental conditions (single-contrast or multi-contrast experiments).
Blinding	No blinding was performed, although all results were analyzes as within-session or within-mouse.

Reporting for specific materials, systems and methods

We require information from authors about some types of materials, experimental systems and methods used in many studies. Here, indicate whether each material, system or method listed is relevant to your study. If you are not sure if a list item applies to your research, read the appropriate section before selecting a response.

Materials & experimental systems

n/a	Involved in the study	
☒	☐	Antibodies
☒	☐	Eukaryotic cell lines
☒	☐	Palaeontology
☐	☒	Animals and other organisms
☒	☐	Human research participants
☒	☐	Clinical data

Methods

n/a	Involved in the study	
☒	☐	ChIP-seq
☒	☐	Flow cytometry
☒	☐	MRI-based neuroimaging

Animals and other organisms

Policy information about studies involving animals; ARRIVE guidelines recommended for reporting animal research

Laboratory animals	Male C57BL/6J mice were obtained from The Jackson Laboratory and used for imaging experiments between 4-7 months of age.
Wild animals	This study did not involve wild animals.
Field-collected samples	This study did not involve field-collect samples.
Ethics oversight	All experimental procedures were approved by the Harvard Medical School Institutional Animal Care and Use Committee (IACUC) and were performed in compliance with the Guide for Animal Care and Use of Laboratory Animals and with the Harvard Medical School Standing Committee on Animals.

Note that full information on the approval of the study protocol must also be provided in the manuscript.
